# Miltirone enhances the chemosensitivity of gastric cancer cells to cisplatin by suppressing the PI3K/AKT signaling pathway

**DOI:** 10.3389/fphar.2025.1553791

**Published:** 2025-04-07

**Authors:** Yiping Wang, Hang Lv, Li Shen, Zhe Chen

**Affiliations:** ^1^ Laboratory of Digestive Pathophysiology of Zhejiang Province, The First Affiliated Hospital of Zhejiang Chinese Medical University, Hangzhou, China; ^2^ Institute of Basic Theory of TCM, China Academy of Chinese Medical Sciences, Beijing, China

**Keywords:** gastric cancer, miltirone, combination therapy, cisplatin, PI3K/Akt signaling pathway

## Abstract

**Background:**

Gastric cancer (GC) is one of the most common malignant tumors with poor survival. Although cisplatin is a first-line chemotherapy drug for GC, it still has the potential to develop drug resistance and side effects. Miltirone, extracted from Chinese herb Salvia miltiorrhiza Bunge, has been reported to significantly inhibit some types of cancer. However, its effects on GC have not been studied, the possible anti-tumor effects of miltirone in combination with cisplatin in GC patients have not been explored.

**Materials and methods:**

Human GC cell lines AGS, HGC27, MKN45 and MGC803 cells were treated with miltirone and cisplatin individually or combinatorially. Cell proliferation assay, flow cytometric assay, colony formation assay and Western blot were employed to evaluate the cytotoxic effects under these treatments. Wound healing and transwell assays were used to examine the effects of miltirone and/or cisplatin on GC cell migration and invasion. RNA-seq analysis was used to determine miltirone’s potential target genes in AGS cells. GO analysis and molecular docking assay were used to determine the pathways affected by miltirone. Next, we examined changes in the selected pathway proteins. The *in vivo* animal model was verified the results of the *in vitro* experiments.

**Results:**

Miltirone inhibited cell growth, migration, and invasion, as well as induced apoptosis in GC cells. In combinatorial treatments, miltirone synergistically enhanced cytotoxicity of cisplatin in GC cells. Moreover, the expression levels of 606 genes appeared to be significantly modulated by miltirone via RNA-seq analyses, and PI3K/AKT signaling pathway was found to refer to miltirone activity. Furthermore, miltirone together with cisplatin treatment significantly reduced the expression levels of p-PI3K, p-Akt, p-mTOR, while the total levels of PI3K and Akt remained unchanged. In addition, compared with the control group, the tumors growth was significantly suppressed in groups treated with the two agents alone or in combination, and even more so in the combination group *in vivo*.

**Discussion:**

Miltirone inhibited the proliferation of GC cells and significantly potentiates the anticancer activities of cisplatin by downregulating the PI3K/AKT signaling pathway. Combination therapy of miltirone and cisplatin represents a novel potential treatment of gastric cancer.

## 1 Introduction

Although the incidence of, and mortality rate from, gastric cancer (GC) has declined in recent years, GC remains a global health problem. Nowadays, GC is the fifth most commonly diagnosed cancer and the fifth most common cause of cancer-related death globally. Nearly 1 million people are newly diagnosed worldwide each year (Bray et al., 2024). The prognosis is poor. Many GCs are of advanced stage at the time of initial diagnosis. The 5-year survival rate is less than 10% (Yang et al., 2020). Currently, the preferred GC treatment is comprehensive surgery, but chemotherapy for postoperative and advanced GC. The classical chemotherapeutic agents used in clinical practice include 5-fluorouracil (5-Fu), cisplatin (also termed cDDP), paclitaxel, and epothilone (Otaegi-Ugartemendia et al., 2022). However, the development of GC resistance to conventional chemotherapeutic agents is a major obstacle to effective chemotherapy. Therefore, new drugs that improve the sensitivity to chemotherapeutic agents are urgently required.

Salvia miltiorrhiza Bunge, (also known as danshen), is a traditional Chinese medicine (TCM) commonly used to treat cardiovascular diseases. Certain bioactive compounds extracted from danshen, such as tanshinone IIA, tanshinone, and phenolic acids, have been developed as commercial drugs (Li et al., 2018). In recent years, increasing evidence has suggested that Salvia miltiorrhiza Bunge could serve as an effective adjuvant in cancer treatment (Ansari et al., 2021; Jia et al., 2024).

**FIGURE 1:**
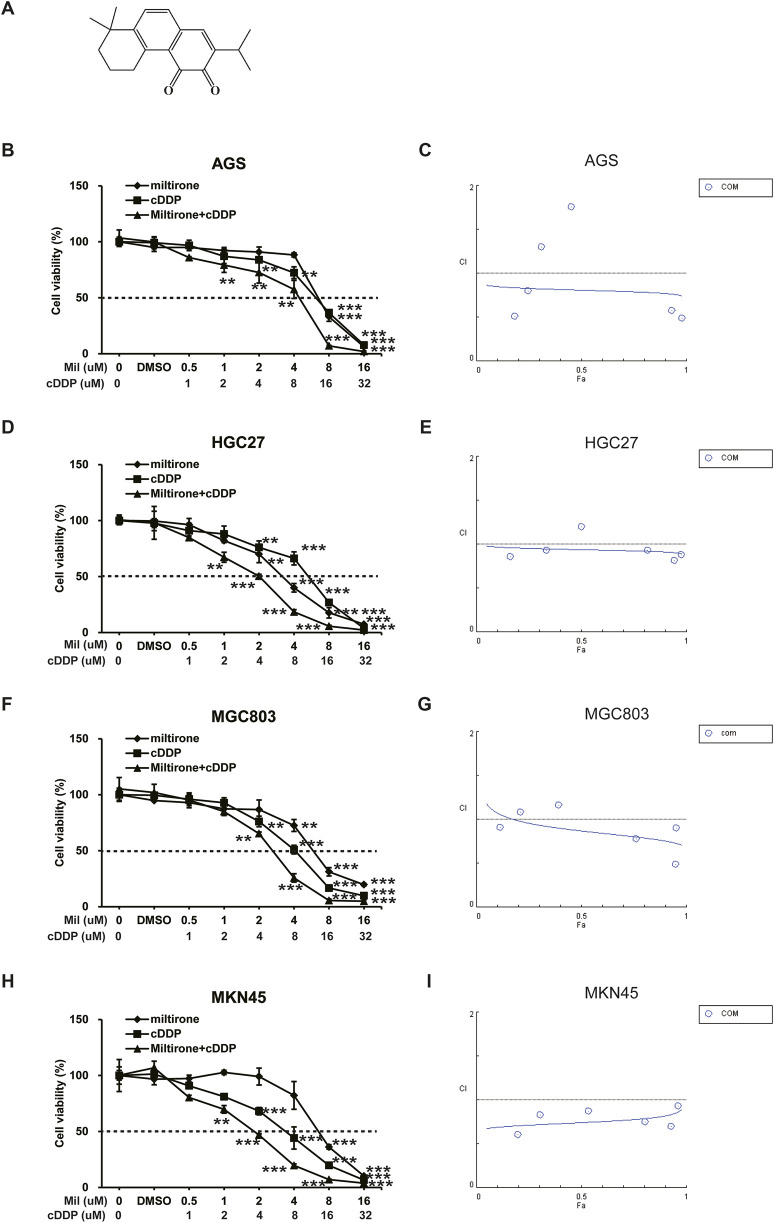
The inhibitory effect of miltirone, cisplatin, and combination therapy on the proliferation of gastric cancer cells. **(A)** Chemical structure of miltirone. Miltirone, cisplatin, and combination treatment inhibited the proliferation of gastric cancer cells as shown by decreased cell viability. The drug concentration-cell viability curve was generated as a percentage of viable cells **(B, D, F, H)**. The synergistic effect between miltirone and cisplatin is represented by a Fa-CI diagram **(C, E, G, I)**. The above data are from three replicate experiments (mean ± SD). Compared with the control group, *p < 0.05, **p < 0.01 or ***p < 0.001.

Miltirone, a derivative of phenanthrenequinone (C_19_H_22_O_2_), has been isolated from the root of Salvia miltiorrhiza Bunge (Figure 1A). Studies have shown that miltirone seemed to exhibit many pharmacological activities, including anti-inflammatory, anti-cancer, and antileukemic actions (Wang et al., 2016; Zhou et al., 2016; Wang et al., 2017). Miltirone exerted anti-cancer activity by inhibiting cell growth and the induction of apoptosis by various signaling pathways. Miltirone affected the generation of mitochondrial reactive oxygen species and regulated the expression of various proteins including p53, the mitogen-activated and extracellular signal-regulated kinase (MEK), and extracellular protein kinases 1/2 (ERK1/2) (Zhou et al., 2015; Zhu, 2018; Zhang et al., 2020). However, the effect of miltirone on GC has not been studied, and the molecular mechanism of the anticancer effect remains to be elucidated.

In the time since discovery of the anticancer activity of cisplatin more than 50 years ago, cisplatin has become one of the most effective and widely used chemotherapeutic agents for treatment of a variety of solid cancers, including ovarian, cervical, bladder, lung, and gastric cancers, and several other cancers (Hamaya et al., 2023; Li et al., 2023; Zhang et al., 2022). Cisplatin enters cells via passive diffusion or through copper transporters and binds to DNA to form DNA-cisplatin adducts, then lead to DNA damages and DNA replication ceases (Ghosh, 2019). The cisplatin adducts are often resistant to DNA repair, persistent DNA damage promotes activation of apoptotic related proteins, including the c-Abl tyrosine kinase, JNK, p38 mitogen-activated protein kinase (MAPK), the oncogene P53, Bax (Jayson et al., 2014; Branch et al., 2000), also induces oxidative stress and impairs mitochondrial respiratory function, ultimately triggers apoptosis of cancer cells (Liu et al., 2023). However, the efficacy of cisplatin chemotherapy is greatly compromised by serious side-effects, the development of drug resistance, and relapse. For instance, mechanisms such as impaired damage recognition, overexpression of HER-2/neu, activation of the PI3K/AKT pathway, loss of p53 function, upregulation of the anti-apoptotic protein Bcl-2, and disruption of caspase activation can collectively contribute to the failure of cisplatin-induced apoptosis in tumor cells (Dasari and Tchounwou, 2014; Gandin et al., 2023). Such clinical limitations have prompted researchers to test cisplatin-based combination therapies. First-line treatment with S-1 plus cisplatin significantly prolonged the median overall survival and progression-free survival of patients with advanced GC (Koizumi et al., 2008). To date, no report has explored possible anti-tumor effects of miltirone in combination with cisplatin in GC patients, nor the underlying mechanisms of any such effects.

In this study, we investigated the effects of a combination of miltirone and cisplatin on GC cells in terms of regulation of PI3K/AKT signaling. In addition, we analyzed the *in vivo* effects of the combination of miltirone and cisplatin using a GC xenograft model. Our results showed that miltirone significantly increased the sensitivity of GC to cisplatin, and miltirone may thus serve as a novel GC treatment.

## 2 Materials and methods

### 2.1 Cells and reagents

Human gastric cancer cells including AGS, HGC27, MGC803 and MKN45 cell line, and one normal gastric mucosal epithelial cell line (GES-1) were purchased from the Cell Bank of the Chinese Academy of Science (Shanghai, China), and all cells were authenticated by STR profiling. Cells were cultured in RPMI-1640 (Gibco, Nuoyang Biotech Co., Ltd., Hangzhou, China) supplemented with 10% fetal bovine serum (Hyclone) in a humidified atmosphere of 5% CO2 at 37°C.

Miltirone was purchased from Chem Faces (Hubei, China) and initially dissolved in dimethyl sulfoxide (DMSO) to obtain a 20 mM stock solution. Cisplatin was purchased from MedChemExpress and dissolved in water at stock concentrations of 1 mM. Both of the stock solutions were stored at −20°C, fresh dilutions in medium were made before use. PI3K inhibitor LY294002 and PI3K activator recilisib from MedChemExpress (USA).

Fetal bovine serum (FBS), 0.25% trypsin containing EDTA was obtained from Gibco (USA). Cell Counting Kit-8 (CCK-8) was purchased from DoJinDo (Japan).

### 2.2 Cell proliferation assay

CCK-8 assay was performed to determine the cytotoxicity of miltirone, or/and cislatin. In briefly, Cells were plated in 96 wells plate (5000 cells per well) and treated by the indicated concentration of drugs for 48 h, Cell viability was assayed by CCK8 kit according to the manufacturer’s manual. Absorbance at 450 nm was measured using a Thermo Varioskan Flash. Alternatively, the Edu Cell Proliferation Kit (Epizyme) was used according to the manufacturer’s instructions. Briefly, cells cultured in 24-well plates were pulsed with Edu before fixation and subsequent detection. The cell nuclei were stained with DAPI. Stained cells were imaged using a Leica SP8 confocal fluorescence microscope.

### 2.3 Colony formation assay

Cells were spread in 24-well plate and cultured with drugs at specific concentration. After 10 days, the cells were fixed with methanol for 5 min and stained with 0.1% crystal violet, then photographed under a microscope. The percentage of area covered by cells per view was calculated with ImageJ.

### 2.4 Western blotting

Total proteins were prepared from cultured cell samples by RIPA buffer (Beyotime, China) with proteases and phosphatases inhibitor cocktail (PhosStop Roche). Equal amounts of proteins were separated on SDS-PAGE and transferred to polyvinylidene difluoride (PVDF) membranes (Life Sciences). The blots were scanned using ChemiDoc XRSt Imaging System (Bio-Rad). Primary antibodies used include: Caspase-3 (CST #9662S 1:1000), cleaved Caspase-3 (CST #9661S 1: 1000), PARP(CST #9542 1:1000), E-cadherin (CST #3195 1:1000), snail (CST #3879 1:1000), vimentin (CST #5741S 1:1000), p-Akt (CST #4060 S 1:1000), Akt (CST #4691 S 1: 1000), p-PI3K (CST #13857 1:1000), PI3K (CST #4249S 1: 1000), mTOR (abcam # ab32028 1:2000), p-mTOR (abcam #ab109268 1:2000), β-actin (CST #4976 1:1000).

### 2.5 Flow cytometry

Apoptosis was detected with FITC Annexin V Apoptosis Detection Kit (BD Bio-sciences). Briefly, cells were treated with drugs at indicated concentration for 48 h. Cells were collected and washed twice with cold phosphate buffered saline (PBS). Cells were then resuspended with 100 µL of 1× binding buffer, 5 μL of PE Annexin V and 5 μL of 7-AAD Viability staining solution were added and reacted for 10 min away from light. Finally, 400 µL of 1× binding buffer was added before the fluorescence of cells was measured with NovoCyte Flow Cytometer (ACEA Biosciences, USA), the apoptosis rates were analyzed with NovoExpress^®^ software (1.4.1, ACEA Biosciences, USA).

### 2.6 Wound healing assay

Wound healing assays was performed by using the ibidi Culture–Insert 4 Well. 5 × 104 cells were seeded onto each well, and the Culture–Insert 4 Well was gently removed with a sterile tweezer after cells grew to 90% confluence. The cells remained were washed three times with PBS, then culture with fresh serum-free medium containing indicated drugs. The migrating cells were photographed under a microscope at the indicated times. The distance of cell migration was analyzed using ImageJ.

### 2.7 Invasion assay

Transwell filter (8.0 μm pore membranes, Corning) was precoated with a thin matrigel matrix (BD Biosciences, Bedford, Massachusetts). 4 × 104 cells suspended in serum-free medium were seeded into the upper compartment, complete medium containing 10% FBS was added into the lower compartment. The cells on the upper compartment were stained with crystal violet after treated with indicated drugs for 48 h, the stained cells were counted under a microscope. Each experiment was performed in triplicate.

### 2.8 RNA-seq analysis

The AGS cells were treated with miltirone (6 µM) or DMSO for 48 h. Cells were collected and washed with PBS twice. The RNA of each group was extracted, the quantity and purity of the total RNA were assessed using a NanoDrop ND-1000 (NanoDrop, Wilmington, DE, USA), and the integrity of the RNA was evaluated using a Bioanalyzer 2100 (Agilent, CA, United States). Subsequently, the qualified RNA was selected for amplification and establishment of cDNA library. The library was sequenced using the illumina Novaseq TM 6000 (LC Bio Technology CO., Ltd. Hangzhou, China). The differential gene expression analysis was carried out by FPKM value with p < 0.05 and | log2 (fold change) | ≥ 4 as a confidence threshold. Bioinformatic analysis was executed using the OmicStudio tools accessible at https://www.omicstudio.cn/tool.

### 2.9 Molecular docking

The three-dimensional structure models of P13K and AKT were obtained from the PDB protein database (https://www.rcsb.org/) in PDB format. The three-dimensional structure models of the substrates were drawn using ChemDraw 20.0 software (CambridgeSoft, Cambridge, United Kingdom). The three-dimensional structures were processed (water removal, hydrogen addition, protonation) using VMD (https://www.ks.uiuc.edu/Research/vmd/). The processed models were subjected to molecular docking using AutoDockTools-1.5.7 software. All models and molecular docking results were visualized and analyzed using PyMOL (http://www.pymol.org/).

### 2.10 Xenograft tumor models

AGS cells (5 × 10^6^) were injected subcutaneously to unilateral axilla of nude mice at the age of 4–5 weeks. When the tumor grew to about 100 mm^3^, the mice were randomly divided into 4 groups. Each group was dosed every other day via intravenous injection with the equal volumes of vehicle (saline), miltirone (10 mg/kg), cisplatin (5 mg/kg), miltiron (10 mg/kg) and DDP (5 mg/kg). Tumor volume was measured every other day and was calculated with the following formula: V=(length*width*width)/2. The anti-tumor rate (%) was calculated using the following relationship: anti-tumor rate (%) = (1-ATWexp/ATWcon) * 100%. Where ATWexp and ATWcon represent the average tumor weight of the treatment group and control group, respectively. All animals were sacrificed after 18 days of drug exposure, the tumors, heart, liver, spleen, lungs, kidneys, and intestines were collected for histological analysis.

### 2.11 Statistical analysis

Statistical comparisons were performed using GraphPad Prism version 5.0 (GraphPad Software, San Diego). Each set of experiment was repeated at least three times, and the data were presented as mean ± standard deviation (SD). One-way ANOVA was used for comparison of tumor volume measured at different times between the groups, and differences between experimental and untreated control groups were analyzed using Student’s t-test. P < 0.05 or less was considered statistically significant.

## 3 Results

### 3.1 The combination of miltirone and cisplatin synergistically inhibits GC cell proliferation

GC cell lines, including AGS, HGC27, MGC803 and MKN45 cells, were used to explore the anti-cancer effects of miltirone and/or cisplatin. Both miltirone and cisplatin inhibited GC cell proliferation in a dose-dependent manner. Over 48 h of treatment, the IC50 values of the two compounds in these 4 cell lines were 6.87, 3.41, 6.39 and 7.48 μM for miltirone and 12.91, 11.14, 8.49 and 5.83 μM for cisplatin, respectively. Subsequently, AGS, HGC27, MGC803 and MKN45 cells were treated with the indicated concentrations of both miltirone and cisplatin for 48 h. Compared to groups treated with one drug alone, the combination of miltirone and cisplatin more effectively inhibited cell proliferation (Figures 1B, D, F, H). A combination index (CI) was calculated to determine whether miltirone and cisplatin combination therapy was synergistic, additive, or antagonistic. The miltirone and cisplatin combination CI was <1, indicating synergistic inhibition of GC cell proliferation after 48 h of treatment (Figures 1C, E, G, I). In addition, we carried out the experiment with GES-1. GES-1 cells were used to explore the anti-cancer effects of miltirone and/or cisplatin at indicated concentration. As shown in Supplementary Figure S1, both miltirone and cisplatin inhibited GC cell proliferation in a dose-dependent manner. Over 48 h of treatment, the IC50 values of the two compounds in GES-1 were 8.17 μM for miltirone and 14.45 μM for cisplatin, respectively. The IC50 value of GES-1 was much higher than gastric cancer cell lines, suggesting that miltirone could specifically inhibit the growth of gastric cancer cells. Moreover, the miltirone and cisplatin combination CI was <1 in GES-1 cells, indicating synergistic inhibition of cell proliferation.

**FIGURE 2:**
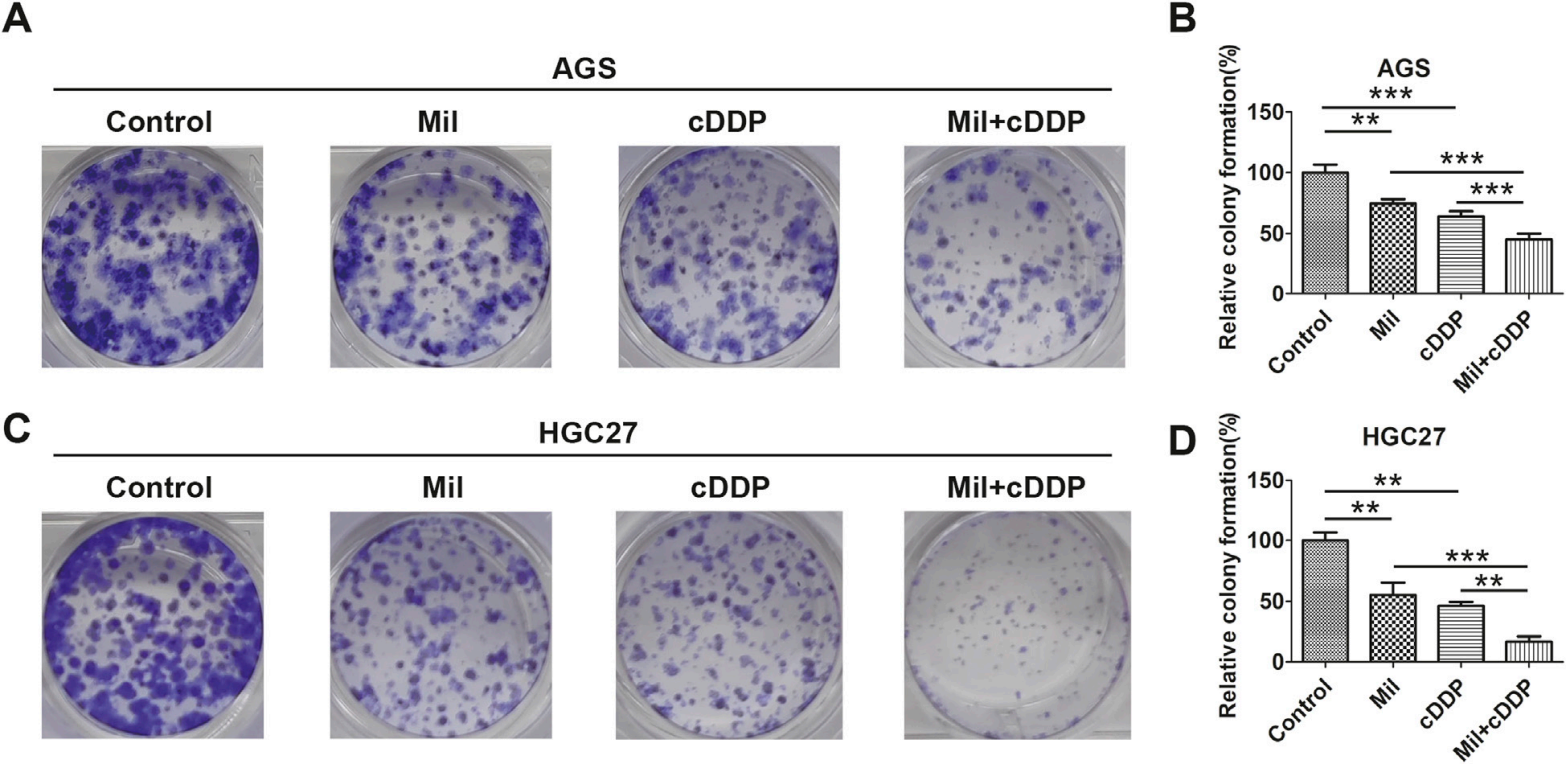
Miltirone enhances the role of cisplatin in gastric cancer cells. **(A)** AGS and **(C)** HGC27 cells were treated with cisplatin (6 μM), miltirone (3 μM) alone, or the combination of cisplatin and miltirone for up to 10 days,the colony formation ability was determined. **(B, D)** Statistical analysis of the colonies in groups of AGS and HGC27 cells treated with drugs at each specified concentration. Data are expressed as (mean ± SD); n = 3; Differences between results for the control group or combined drug treated groups and only one drug treated groups are indicated using p-values, *p < 0.05, **p < 0.01, and ***p < 0.001. cDDP cisplatin, Mil, miltirone.

A colony formation assay was next used to determine the anti-cancer effects of miltirone and cisplatin on GC cells. After treatment with miltirone and/or cisplatin for 10 days, AGS and HGC27 colony formation was inhibited by each compound alone, but the effects were more significant when miltirone was combined with cisplatin (Figures 2A–D). Collectively, these results suggested that miltirone might enhance the tumor-suppressive bioactivity of cisplatin in GC patients.

### 3.2 The combination of miltirone and cisplatin induces apoptosis of GC cells

**FIGURE 3:**
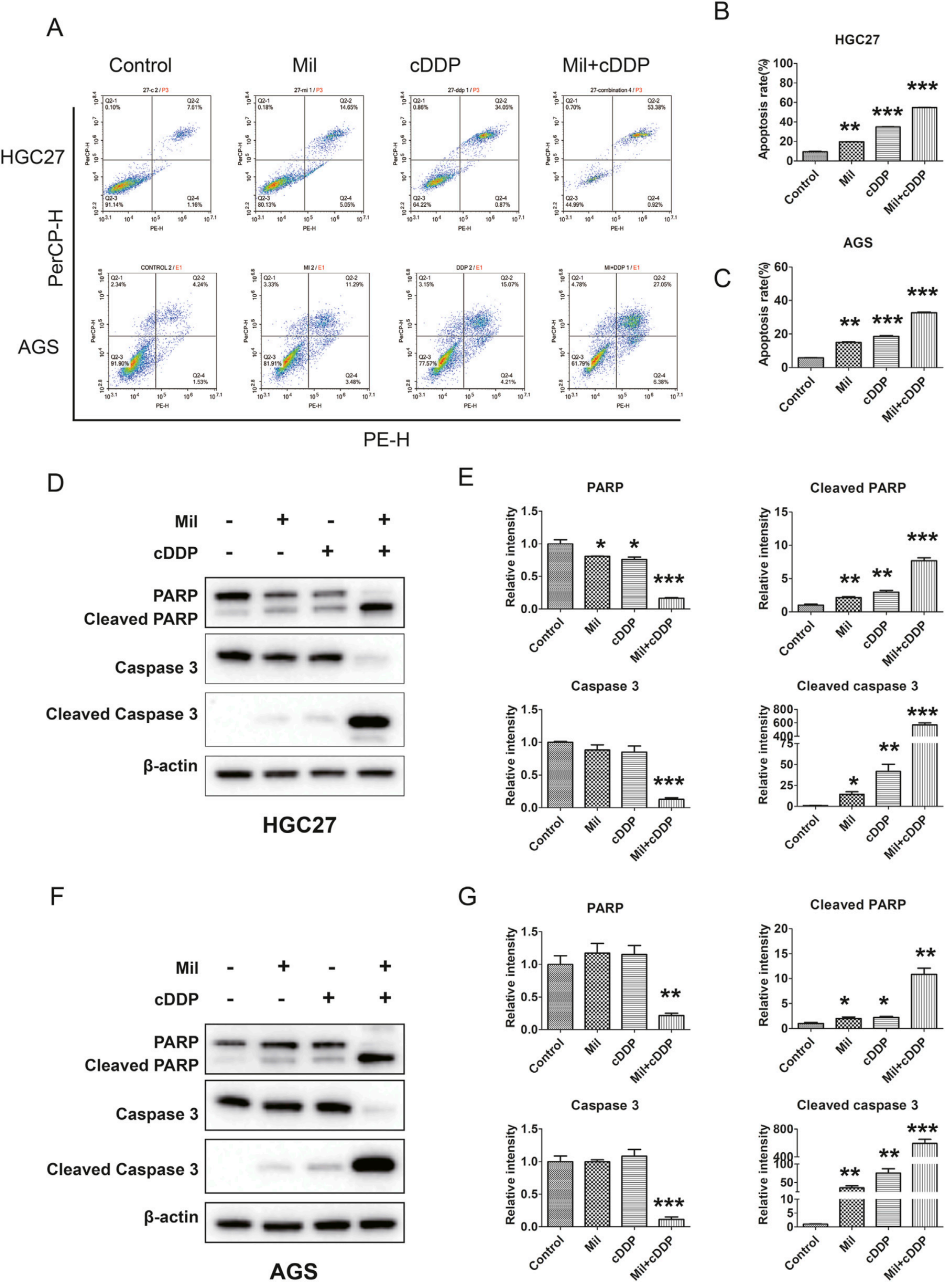
Effect of miltirone and cisplatin alone or in combination on apoptosis of gastric cancer cells. **(A)** HGC27 and AGS cells were treated with either miltirone (3 μM) or cisplatin (6 μM) alone, or cisplatin and miltirone combined for 48 h, the cell apoptosis was analyzed by flow cytometry. All data are shown as the (mean ± SD) of three independent experiments. **(B, C)** Quantitative analysis of apoptotic cells. The percent-age of apoptotic cells was represented by a bar diagram. Data are presented as the mean ± SD of three independent experiments. Compared with the control group, *p < 0.05, **p < 0.01, and ***p < 0.001. **(D)** HGC27 and **(F)** AGS cells were treated with either miltirone (3 μM) or cisplatin (6 μM) alone, or cisplatin and miltirone combined for 48 h, and cell lysates were subjected to Western blot analysis with PARP, cleaved PARP, caspase3 and cleaved caspase 3 antibodies. The relative gray values of related proteins in HGC27 **(E)** and AGS **(G)** were measured using ImageJ. All data are shown as the (mean ± SD) of three independent experiments. Compared with the control group, *p < 0.05, **p < 0.01, and ***p < 0.001. cDDP cisplatin, Mil, miltirone.

Given the anti-proliferative effects of miltirone and cisplatin, we further determined the effects of miltirone and/or cisplatin on apoptosis of GC cells. AGS and HGC27 GC cells were treated with the indicated concentrations of miltirone and/or cisplatin and subjected to flow cytometry after staining with PE Annexin V/7-AAD. Both miltirone and cisplatin induced apoptosis of GC cells, and the combined treatment significantly enhanced apoptosis, as compared to that afforded by either compound alone (Figures 3A–C). Western blotting revealed that the levels of apoptotic markers, including cleaved caspase-3 and cleaved poly-ADP ribose polymerase (PARP), increased after miltirone and/or cisplatin treatment (Figures 3D–G). Together, these data suggest that miltirone triggered apoptosis that repressed the proliferation of GC cells, and that miltirone also enhanced apoptosis induced by cisplatin.

### 3.3 The combination of miltirone and cisplatin synergistically inhibits GC migration and invasion

**FIGURE 4:**
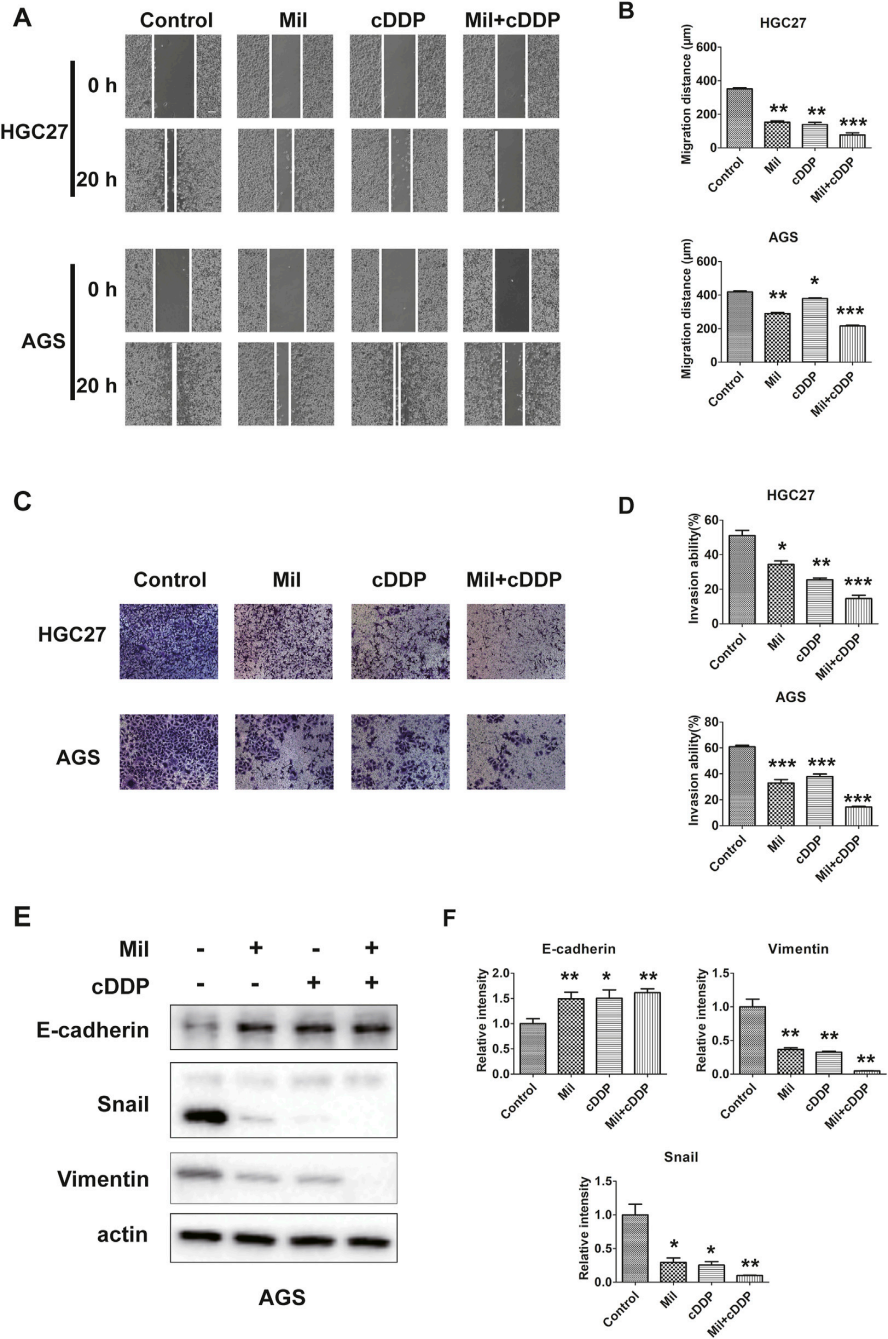
Miltirone and cisplatin inhibit the migration and invasion ability of gastric cancer cells. **(A)**wound healing and **(C)** transwell assay were performed using AGS and HGC27 cells treated with either miltirone (3 μM) or cisplatin (6 μM) alone, or cisplatin and miltirone combined for 48 h (scale bar 100 μm). The histogram describes the average migration distance **(B)** and the percentage of invaded cells **(D)**, respectively. Data are presented as the mean ± SD of three independent experiments. Compared with the control group, *p < 0.05, **p < 0.01, and***p < 0.001. **(E)** The expression of EMT-related proteins, including E-cadherin, snail, vimentin, in AGS were detected by Western blotting assay. The relative gray values of related proteins in AGS **(F)** were measured using ImageJ. All data are shown as the (mean ± SD) of three independent experiments. *p < 0.05, **p < 0.01, and***p < 0.001 relative to the control group. DDP cisplatin, Mil, miltirone.

We next examined whether the combination of miltirone and cisplatin affected the migration and invasion of GC cells. AGS and HGC27 cells were treated with miltirone and/or cisplatin and then subjected to wound-healing and transwell migration assays. Both miltirone and cisplatin alone significantly decreased the number of GC cells that invaded through matrigel, and the migration distance. Compared to either compound alone, combined therapy was associated with the smallest migration distance and the lowest invaded cell numbers (Figures 4A–D). We next examined the levels of epithelial–mesenchymal transition (EMT) proteins. Miltirone and cisplatin alone increased the E-cadherin level and reduced the snail and vimentin levels. Compared to either compound alone, the combined treatment significantly downregulated the expression of snail and vimentin, and upregulated the E-cadherin level (Figures 4E, F). Together, these results suggest that miltirone and cisplatin synergistically inhibited the migration and invasion of GC cells.

### 3.4 Miltirone enhances the sensitivity of GC cells to cisplatin by modulating the PI3K/AKT signaling pathway

**FIGURE 5:**
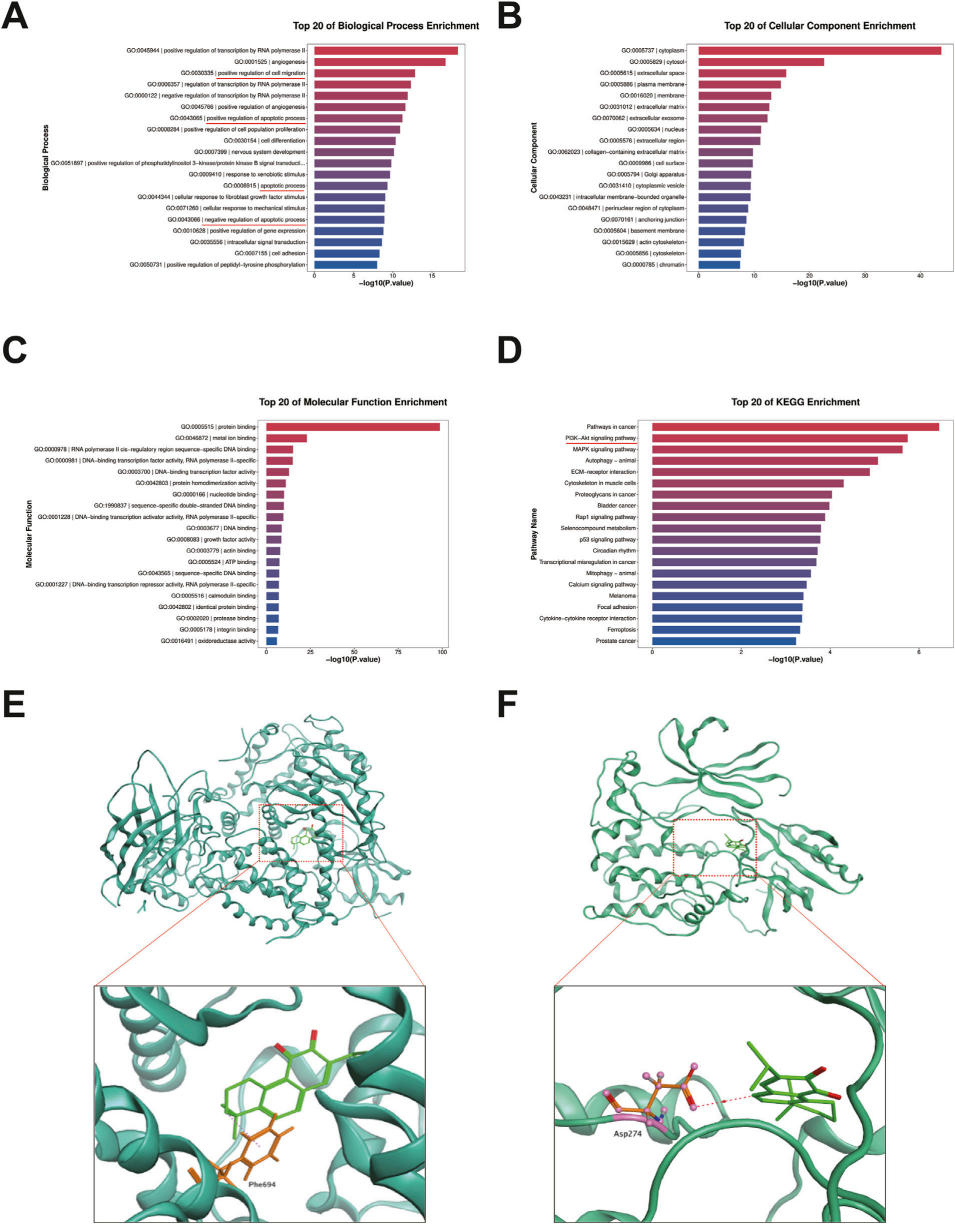
GO, KEGG pathway and molecular docking analysis of miltirone. GO and KEGG pathway analysis of miltirone-treated AGS cells. The Top 20 signaling pathways of miltirone in GO-BP pathway analysis **(A)** GO-CC **(B)** GO-MF **(C)** the Top 20 signaling pathways of miltirone in KEGG pathway analysis **(D)**. Molecular docking of miltirone to PI3K **(E)** and AKT **(F)** protein.

To investigate the potential molecular mechanisms by which miltirone induced apoptosis of, and inhibited migration by, GC cells, we used RNA-seq to profile differentially expressed genes in AGS cells treated with miltirone (5 μM) for 48 h. Finally, the expression levels of 606 genes appeared to be significantly modulated by miltirone (fold change ≥4), including 94 upregulated genes and 512 downregulated genes (Supplementary Table S1). GO and KEGG analyses were used to characterize the canonical pathways. As shown in Figures 5A–D, the GO pathways of “apoptotic process”, “positive regulation of cell migration”, and “PI3K/AKT signaling” were enriched, suggesting that a cluster of miltirone-regulated genes were related to the PI3K/AKT pathway.

### 3.5 Docking of miltirone with targeted molecules

As miltirone was predicted to enhance the sensitivity of GC cells to cisplatin via modulation of the PI3K/AKT signaling pathway, we investigated possible binding of miltirone to key proteins of the PI3K/AKT signaling pathway. The Molecular Operating Environment (MOE, 2019) was used to explore molecular docking of miltirone with PI3K, AKT, and mTOR. The results showed that miltirone could bind to PI3K and AKT via both hydrogen-bonding and hydrophobic interactions (Figures 5E, F) with binding energies of −6.52 and −6.50 kcal/mol, respectively.

### 3.6 Combined treatment with miltirone and cisplatin reduces the activity of the PI3K/AKT signaling pathway in GC cells

**FIGURE 6:**
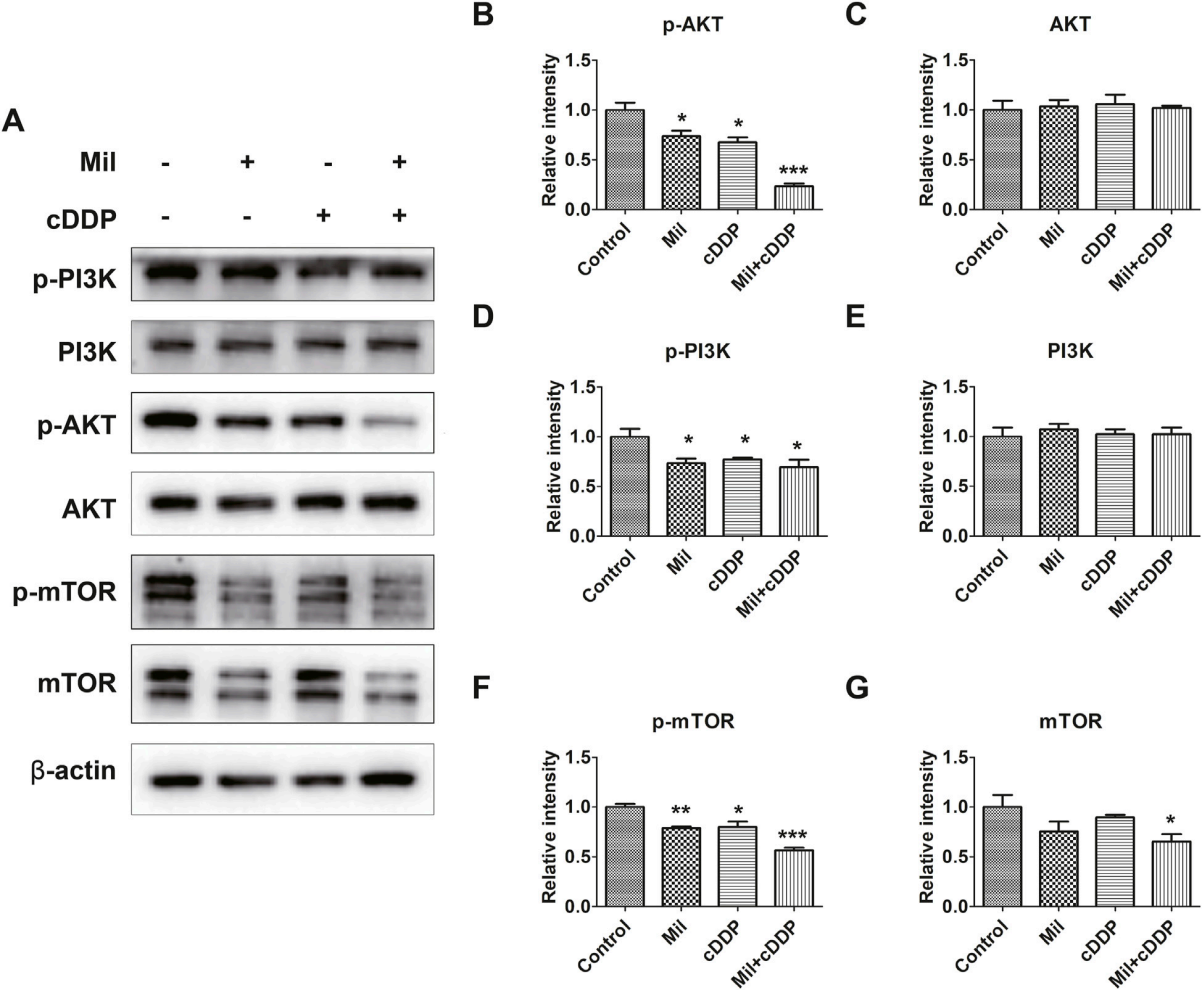
Miltirone, cisplatin, and the combination of the two drugs inhibit the PI3K/AKT signaling pathway in AGS cells. **(A)** Protein expression levels of p-PI3K, PI3K, p-Akt, Akt, mTOR, p-mTOR and β-actin in AGS cells treated with either miltirone (3 μM) or cisplatin (6 μM) alone, or cisplatin and miltirone for 48 h **(B–G)** The histogram describes the relative gray values of related proteins measured using ImageJ. All data are shown as the (mean ± SD) of three independent experiments. *p < 0.05, **p < 0.01, and***p < 0.001 compared with the control group. cDDP, cisplatin, Mil, miltirone.

Given that the PI3K/AKT signaling pathway might be associated with the biological functions of miltirone, we subjected AGS cells to Western blot analysis after single and/or double treatment. We exposed AGS cells to miltirone and/or cisplatin for 48 h and used Western blotting to assess the expression of proteins related to the PI3K/AKT signaling pathway. The expression levels of p-PI3K, p-Akt, and p-mTOR in the single-agent groups were suppressed compared to the control group, but the total Akt, PI3K, and mTOR levels remained unchanged (Figures 6A–G). Moreover, compared to the single-agent groups, the expression levels of p-PI3K, p-Akt, and p-mTOR in the miltirone/cisplatin combined treatment group were lower (Figures 6A–G), suggesting that the effects were more significant in that group.

### 3.7 The PI3K/AKT signaling pathway mediates the effect of miltirone on proliferation in GC cells

**FIGURE 7:**
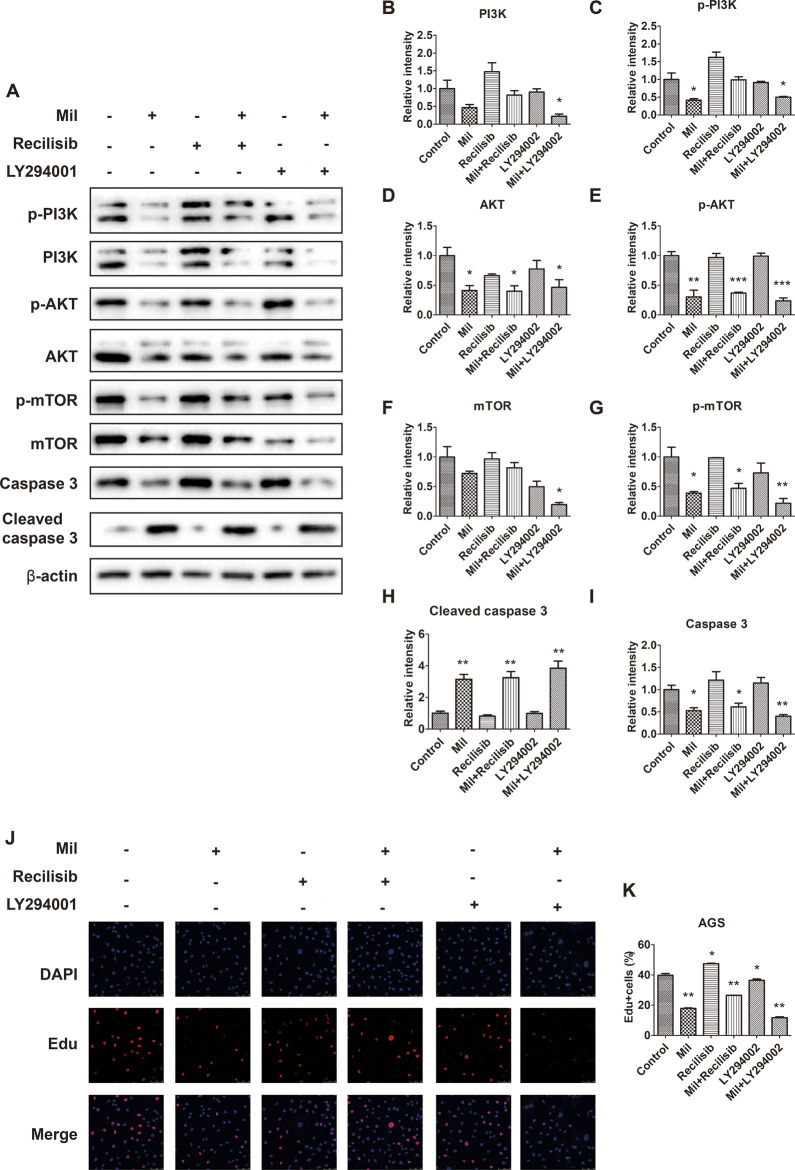
The PI3K/AKT signaling pathway mediates the inhibition of miltirone on proliferation in AGS cells. **(A)** Protein expression levels of p-PI3K, PI3K, p-Akt, Akt, mTOR, p-mTOR, caspase 3, cleaved caspase 3 and β-actin in AGS cells treated with either miltirone (3 μM), PI3K activator recilisib (10 μM) or PI3K inhibitor LY294002 (10 μM) alone, or recilisib/LY294002 and miltirone for 48 h **(B–I)** The histogram describes the relative gray values of related proteins measured using ImageJ. All data are shown as the (mean ± SD) of three independent experiments. *p < 0.05, **p < 0.01, and***p < 0.001 compared with the control group. Mil, miltirone. **(J)** AGS cells treated with either miltirone (3 μM), PI3K activator recilisib (10 μM) or PI3K inhibitor LY294002 (10 μM) alone, or recilisib/LY294002 and miltirone for 48 h, and DNA synthesis determined using Edu incorporation for 3 h in AGS cells. **(K)** Representative images showing nuclei (DAPI staining, blue) or incorporated Edu (red) were subjected to image analysis to determine comparative DNA synthesis rates (bar = 50 µm). All data are shown as the (mean ± SD) of three independent experiments. *p < 0.05, and **p < 0.01 compared with the control group. Mil, miltirone.

Since miltirone enhances the sensitivity of cisplatin to gastric cancer cells via modulating the phosphorylation levels of key factors in the PI3K/AKT signaling pathway, we further investigated the relationship between miltirone and PI3K/AKT by treating cells with Recilisib, a pathway activator, and LY294002, an inhibitor, at a concentration of 10 μM. The results showed that recilisib attenuated the inhibitory effect of miltirone on the PI3K/AKT signaling pathway. As shown in Figure 7, compared with group treated with miltirone alone, the expression levels of p-PI3K and p-AKT increased after recilisib and miltirone treatment, while p-PI3K, p-AKT and p-mTOR expression decreased in LY294002 together with miltirone treated group. Although recilisib did not affect the expression of apoptotic markers cleaved caspase-3, the combined use of LY294002 and miltirone increased the expression of apoptotic markers cleaved caspase-3. In addition, the results obtained from cell immunofluorescence staining further supported these findings. Compare with miltirone group, recilisib combine with miltirone treatment increased the percentage of Edu-positive cells, while LY294002 decreased in the rates of Edu incorporation. Our results provided direct evidence of the inhibition of miltirone on the PI3K/AKT signaling pathway in gastric cancer cells.

### 3.8 Combined treatment with miltirone and cisplatin synergistically suppresses growth of GC tumor xenografts *in vivo*

**FIGURE 8:**
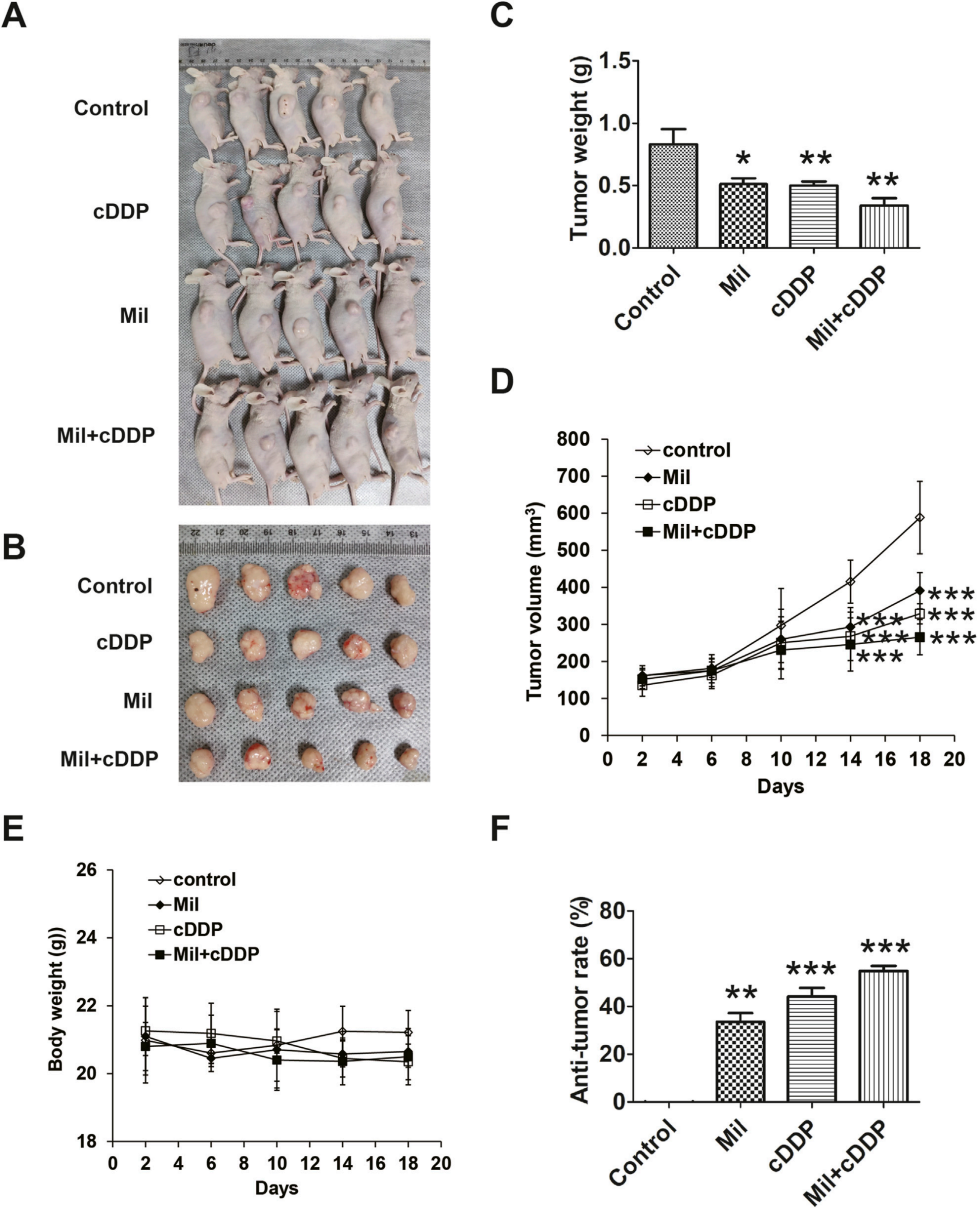
Miltirone combined with cisplatin synergistically suppress tumor growth *in vivo*. AGS cells (5 × 10^6^ cells) were injected subcutaneously into nude mice, and miltirone and/or cisplatin (control group; miltirone treatment group (10 mg/kg); cisplatin treatment group (5 mg/kg); miltirone (10 mg/kg) and cisplatin (5 mg/kg) combined treatment group) were injected intravenously every 2 days after the tumor grew to about 100 mm^3^. **(A)** The transplanted tumors in nude mice. **(B)** The transplantation tumors removed from nude mice. **(C)** Tumor weight and **(D)** Tumor volume was measured on the indicated days. **(E)** Body weight was measured on the indicated days. **(F)** Quantitative analysis of anti-tumor rate was represented by a bar diagram (bar graphs are plotted as the mean ± SD, *p < 0.05, **p < 0.01, and***p < 0.001).

Nude mice bearing AGS-derived tumor xenografts were randomly divided into four groups and treated as follows: Control group (saline); miltirone group (20 mg/kg); cisplatin group (5 mg/kg); and a combined treatment with miltirone (20 mg/kg) and cisplatin (5 mg/kg) group. The effects of the single or combined compounds on tumor growth were observed every other day. As shown in Figure 8, compared to that of the control group, the tumor weight was markedly suppressed in both the miltirone and cisplatin treatment groups. The tumor weight of the combined agents group was significantly less than those of the single treatment groups. As shown in Figure 8F, the tumor suppression rates were 33.56% and 44.17% in the miltirone and cisplatin groups, respectively, but that of the combined group was 54.86%, thus significantly higher (P < 0.05). These results suggest that miltirone/cisplatin combination therapy effectively suppressed tumor growth in GC xenograft mice, and that miltirone could enhance the antitumor effect of cisplatin *in vivo*.

In addition, we assessed the mouse toxicity of miltirone. The body weights did not differ significantly between the miltirone and control groups (Figure 8E). H&E staining revealed no overt morphological change in the vital organs, including the lungs, liver, and kidneys, after miltirone treatment (Supplementary Figure S2), suggesting that miltirone had no toxic effect.

## 4 Discussion

The current treatments for GC patients include surgery, radiotherapy, chemotherapy, and biotherapy. Notably, chemotherapy is an important treatment option for patients with advanced GC. Commonly used chemotherapeutic agents include anti-metabolites, mitotic spindle inhibitors, alkylating agents, and topoisomerase inhibitors (Gielecińska et al., 2023). Platinum-based agents including cisplatin, carboplatin, and oxaliplatin, all of which are alkylating agents, serve as first-line chemotherapeutic drugs and components of treatment regimens for patients with GC (Smyth et al., 2020; Ooki et al., 2022). Cisplatin is a small platinum compound that was initially used for cancer chemotherapy in the late 1970s (Shen et al., 2012). The initial response of GC patients is often promising, but the side-effects, including severe toxicity and drug resistance, markedly reduce later clinical effectiveness (Yamaguchi et al., 2022; Tang et al., 2023). Thus, it is urgent not only to preserve the efficacy of cisplatin in terms of eliminating cancer cells but also to improve quality of life and the therapeutic outcomes.

Increasing evidence has revealed that natural compounds extracted from TCMs may enhance the efficacy of cisplatin or reverse resistance to cisplatin in patients with tumors of various types (Song et al., 2022; Yang et al., 2024). For example, ellagic acid, a natural polyphenolic compound produced by many fruits, selectively and markedly potentiated the anti-cancer activities of cisplatin on hepatocellular carcinoma cells but not normal liver HL-7702 cells (Zhong et al., 2019). Capsaicin, a major pungent ingredient of red pepper, significantly enhanced the effects of cisplatin in terms of osteosarcoma cell apoptosis, pro-survival autophagic induction, cell cycle arrest, and inhibition of invasion both *in vitro* and *in vivo* (Wang et al., 2018).

Miltirone is an active component of Salvia miltiorrhiza Bunge, which exhibits multiple biological and pharmacological activities. Although the curative effects of Salvia miltiorrhiza Bunge have been extensively studied, the bioactive ingredients that are responsible are largely unknown. The main bioactive component of Salvia miltiorrhiza Bunge is tanshinone, which has been traditionally used to treat cardiovascular and cerebrovascular diseases (Li et al., 2020; Wei et al., 2023). Recent pharmacological studies found that several types of tanshinone, including tanshinone IIA, dihydrotanshinone, tanshinone I, and cryptotanshinone, exhibited different inhibitory effects on tumor cells (Jin et al., 2022; Zhang et al., 2023). Tanshinone IIA attenuated the stemness and enhanced adriamycin sensitivity of breast cancer cells by regulating the miR-125b/STARD13 axis (Li et al., 2022). Tanshinone I inhibited proliferation, induced G0/G1-phase arrest, and triggered apoptosis of human hepatocellular carcinoma cells (Liu and Liu, 2020). Cryptotanshinone selectively inhibited the growth and proliferation of HCT116 colorectal cancer cells by activating autophagic signaling mediated by endoplasmic reticulum stress (Fu et al., 2021). A few reports found that miltirone exerted anti-cancer actions, mainly by inhibiting cell growth and inducing apoptosis (Shakeri et al., 2022; Zheng et al., 2024). One study suggested that miltirone induced apoptosis of cisplatin-resistant lung cancer cells (Zhu, 2018), thus indicating that miltirone might enhance the anti-tumor effects of cisplatin. However, the effects of miltirone combined with cisplatin have not yet been reported, nor have the possible mechanisms of such effects.

In this study, the CCK8 assay proved that miltirone and cisplatin suppressed the proliferation of AGS, HGC27, MGC803, and MKN45 GC cells in a dose-dependent manner. Although miltirone exerted a relatively weak inhibitory effect on the proliferation of GC cell lines compared to cisplatin alone, miltirone and cisplatin synergistically reduced cell proliferation to a level lower than that induced by cisplatin alone. Thus, we hypothesized that miltirone might synergistically enhance the effects of cisplatin in terms of GC treatment. Moreover, the normal gastric mucosal epithelial cell line GES-1 demonstrated a higher tolerance to miltirone exposure. While co-treatment with miltirone and cisplatin exhibited a synergistic effect in GES-1 cells, this combined efficacy was only apparent at higher concentrations. These findings suggest that the combination of miltirone and cisplatin is more effective in gastric cancer cells compared to normal gastric mucosal epithelial cells. In the colony-formation and apoptosis assays, compared to cisplatin alone, co-treatment with miltirone and cisplatin was associated with superior inhibitory effects, verifying our assumptions. Moreover, miltirone suppressed GC cell invasion and migration and acted synergistically with cisplatin to inhibit GC invasion and metastasis. The expression levels of proteins that regulate the EMT were consistent with these results. Co-treatment with miltirone and cisplatin downregulated the snail and vimentin levels and upregulated E-cadherin expression. To sum up, miltirone could further induce apoptosis and inhibit the proliferation, invasion, and metastasis of GC cells exposed to cisplatin. Given the synergistic actions of miltirone and cisplatin on GC cells, miltirone may be an effective anti-cancer drug if prescribed as postoperative adjuvant therapy.

The PI3K/AKT signaling pathway plays a central role in the responses of cancer cells to changed pathological and physiological conditions. PI3K is an intracellular lipid kinase that regulates cell proliferation and survival (Engelman, 2009). Akt, an important down-stream target of PI3K, is a serine/threonine kinase linked to GC development and, thus, greatly overexpressed in GC cells (Morgos et al., 2024). In tumors, mTOR, the target of mammalian rapamycin, is often activated by the upstream PI3K/Akt signaling pathway (Zhao et al., 2019). The PI3K/AKT/mTOR pathway regulates cell survival, growth, and metabolism, and is abnormally activated or deregulated in most human cancers; it is associated with anti-apoptosis and pro-survival properties (Fattahi et al., 2020; Kang and Chau, 2020; Baghery Saghchy Khorasani et al., 2021). For example, mTOR hyperactivation was reported in 60%–80% of all gastric adenocarcinomas (Lang et al., 2007; Feng et al., 2008), and inappropriate Akt and p-Akt expression in more than 74% of GC patients (Nam et al., 2003). Therefore, targeting of PI3K/AKT might aid GC treatment. Here, RNA-seq data on miltirone-treated GC cells revealed that the expression levels of many genes encoding components of the PI3K/AKT signaling pathway changed markedly, indicating that PI3K/Akt pathway signaling might be modulated by miltirone in GC cells. AGS cells were treated with miltirone or cisplatin alone, or the combination, and Western blotting used to examine changes in the levels of proteins of the PI3K/AKT signaling pathway. In general, such changes were more significant in cells treated with the combination than the single agents. All of p-PI3K, p-Akt, and p-mTOR were downregulated, suggesting that effects on the PI3K/Akt/mTOR signaling pathway explain, at least in part, why miltirone enhances the sensitivity of GC cells to cisplatin. Furthermore, when GC cells were co-treated with recilisib, a PI3K activator, the inhibitory effects of miltirone on cell proliferation were significantly reduced, as demonstrated by the Edu assay. Conversely, co-treatment with LY294002, a PI3K inhibitor, and miltirone enhanced the suppression of cell proliferation. These findings suggest that miltirone exerts its anti-cancer effects by modulating the PI3K/AKT signaling pathway. Current research highlights a strong correlation between cancer development and inflammatory processes, particularly in gastric cancer (Zeng and Jin, 2022). The progression of gastric cancer encompasses multiple stages, including chronic gastritis, atrophic gastritis, intestinal metaplasia, dysplasia, early gastric cancer, advanced gastric cancer, and late-stage gastric cancer. Some anti-inflammatory therapies showed great efficacy in cancer prevention and treatment (Greten and Grivennikov, 2019; Denk and Greten, 2022). Evidence suggests that miltirone exhibits anti-inflammatory properties (Wang et al., 2017), and the PI3K/AKT pathway plays a regulatory role in inflammatory responses (Schultze et al., 2012; Roy et al., 2023). Whether the anticancer bioactivity of miltirone is related to its anti-inflammatory properties warrants further investigation.

Finally, to verify the effects *in vivo*, we compared tumor growth in mice with GC cell xenografts. Compared with the control group, the growth of xenografted tumors was significantly suppressed in groups treated with the two agents alone or in combination, but more so in the combination group.

## 5 Conclusion

**FIGURE 9:**
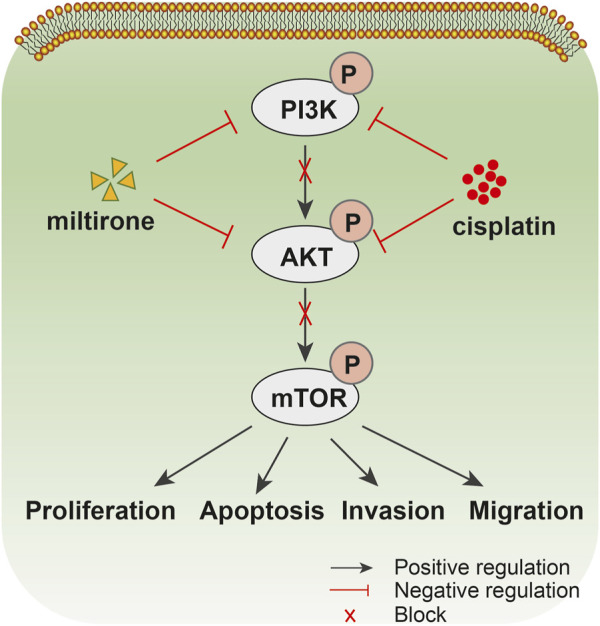
Drug action diagram. Schematic diagram summarizing the proposed model for miltirone enhances the chemosensitivity of gastric cancer cells to cisplatin b via inhibiting PI3K/AKT pathway.

In conclusion, we show that miltirone inhibits the proliferation of GC cells and significantly potentiates the anticancer activities of cisplatin both *in vivo* and *in vitro* by downregulating the PI3K/Akt signaling pathway (Figure 9). Therefore, combined application of miltirone and cisplatin may improve the treatment efficacy of cisplatin-resistant GC patients. Prospective clinical trials are needed in future.

## Data Availability

The original contributions presented in the study are included in the article/Supplementary Material, further inquiries can be directed to the corresponding authors.
