# Supplementary material for: Miltirone enhances the chemosensitivity of gastric cancer cells to cisplatin by suppressing the PI3K/AKT signaling pathway
Source: Front Pharmacol. 2025 Apr 7;16:1553791. doi: 10.3389/fphar.2025.1553791 (PMC12009761; doi:10.3389/fphar.2025.1553791)
Supplement: Supplementary file 1 [file DataSheet1.docx]

Supplementary Material

# Supplementary Data

Supplementary Material includes original images for Western blotting figures and a PDF version that combined cropped protein blot images into the entire membrane

# Supplementary Figures and Tables

## Supplementary Table

Supplementary Table S1, List of differentially regulated genes identified in the miltirone-treated AGS cells compared to that of control AGS cells

| gene_id | gene_name | log2(fc) | regulation |
| --- | --- | --- | --- |
| ENSG00000130513 | *GDF15* | -5.55 | down |
| ENSG00000136244 | *IL6* | -6.29 | down |
| ENSG00000175197 | *DDIT3* | -4.82 | down |
| ENSG00000087074 | *PPP1R15A* | -5.00 | down |
| ENSG00000175592 | *FOSL1* | -4.88 | down |
| ENSG00000116717 | *GADD45A* | -4.42 | down |
| ENSG00000139289 | *PHLDA1* | -5.28 | down |
| ENSG00000138166 | *DUSP5* | -4.78 | down |
| ENSG00000177606 | *JUN* | -4.39 | down |
| ENSG00000173110 | *HSPA6* | -7.65 | down |
| ENSG00000183696 | *UPP1* | -4.55 | down |
| ENSG00000185022 | *MAFF* | -5.04 | down |
| ENSG00000128165 | *ADM2* | -4.81 | down |
| ENSG00000244405 | *ETV5* | -4.16 | down |
| ENSG00000169429 | *CXCL8* | -7.70 | down |
| ENSG00000064300 | *NGFR* | -4.89 | down |
| ENSG00000136167 | *LCP1* | -7.05 | down |
| ENSG00000128965 | *CHAC1* | -4.80 | down |
| ENSG00000167723 | *TRPV3* | -6.40 | down |
| ENSG00000056558 | *TRAF1* | -6.01 | down |
| ENSG00000156510 | *HKDC1* | -5.93 | down |
| ENSG00000187678 | *SPRY4* | -6.63 | down |
| ENSG00000134668 | *SPOCD1* | -4.02 | down |
| ENSG00000104856 | *RELB* | -4.05 | down |
| ENSG00000142173 | *COL6A2* | -4.21 | down |
| ENSG00000095752 | *IL11* | -4.31 | down |
| ENSG00000225217 | *HSPA7* | -8.01 | down |
| ENSG00000290862 | *NMRAL2P* | -4.35 | down |
| ENSG00000293352 | *RPLP0P2* | -8.43 | down |
| ENSG00000148677 | *ANKRD1* | -4.99 | down |
| ENSG00000140297 | *GCNT3* | -4.99 | down |
| ENSG00000117226 | *GBP3* | -4.50 | down |
| ENSG00000156453 | *PCDH1* | -4.51 | down |
| ENSG00000198576 | *ARC* | -4.45 | down |
| ENSG00000138100 | *TRIM54* | -4.17 | down |
| ENSG00000196611 | *MMP1* | -4.87 | down |
| ENSG00000121068 | *TBX2* | -4.19 | down |
| ENSG00000145777 | *TSLP* | -4.59 | down |
| ENSG00000008517 | *IL32* | -4.25 | down |
| ENSG00000116701 | *NCF2* | -4.44 | down |
| ENSG00000170454 | *KRT75* | -9.96 | down |
| ENSG00000048052 | *HDAC9* | -4.05 | down |
| ENSG00000175264 | *CHST1* | -6.48 | down |
| ENSG00000049249 | *TNFRSF9* | -7.44 | down |
| ENSG00000166670 | *MMP10* | -7.93 | down |
| ENSG00000170689 | *HOXB9* | -4.40 | down |
| ENSG00000228509 | *ENSG00000228509* | -6.52 | down |
| ENSG00000135077 | *HAVCR2* | -6.32 | down |
| ENSG00000115423 | *DNAH6* | -4.39 | down |
| ENSG00000263155 | *MYZAP* | -4.21 | down |
| ENSG00000069431 | *ABCC9* | -8.38 | down |
| ENSG00000237330 | *RNF223* | -4.23 | down |
| ENSG00000186642 | *PDE2A* | -5.25 | down |
| ENSG00000285565 | *ENSG00000285565* | -17.00 | down |
| ENSG00000163219 | *ARHGAP25* | -8.16 | down |
| ENSG00000171903 | *CYP4F11* | -7.99 | down |
| ENSG00000155066 | *PROM2* | -6.03 | down |
| ENSG00000104892 | *KLC3* | -4.54 | down |
| ENSG00000007314 | *SCN4A* | -5.05 | down |
| ENSG00000147614 | *ATP6V0D2* | -7.69 | down |
| ENSG00000159899 | *NPR2* | -7.66 | down |
| ENSG00000163216 | *SPRR2D* | -15.73 | down |
| ENSG00000198203 | *SULT1C2* | -5.03 | down |
| ENSG00000188112 | *C6orf132* | -4.17 | down |
| ENSG00000124882 | *EREG* | -6.60 | down |
| ENSG00000131737 | *KRT34* | -14.64 | down |
| ENSG00000250033 | *SLC7A11-AS1* | -4.49 | down |
| ENSG00000185467 | *KPNA7* | -4.20 | down |
| ENSG00000163347 | *CLDN1* | -6.71 | down |
| ENSG00000155918 | *RAET1L* | -4.31 | down |
| ENSG00000112559 | *MDFI* | -5.00 | down |
| ENSG00000153132 | *CLGN* | -5.11 | down |
| ENSG00000134259 | *NGF* | -6.97 | down |
| ENSG00000167755 | *KLK6* | -13.91 | down |
| ENSG00000223874 | *TESHL* | -6.84 | down |
| ENSG00000229261 | *ENSG00000229261* | -5.04 | down |
| ENSG00000117152 | *RGS4* | -12.58 | down |
| ENSG00000006606 | *CCL26* | -5.25 | down |
| ENSG00000103647 | *CORO2B* | -12.69 | down |
| ENSG00000165606 | *DRGX* | -12.97 | down |
| ENSG00000146592 | *CREB5* | -7.06 | down |
| ENSG00000164744 | *SUN3* | -5.20 | down |
| ENSG00000230795 | *HLA-K* | -14.52 | down |
| ENSG00000153294 | *ADGRF4* | -6.63 | down |
| ENSG00000140678 | *ITGAX* | -4.51 | down |
| ENSG00000178445 | *GLDC* | -7.81 | down |
| ENSG00000164220 | *F2RL2* | -4.38 | down |
| ENSG00000120217 | *CD274* | -6.14 | down |
| ENSG00000149599 | *DUSP15* | -12.47 | down |
| ENSG00000154133 | *ROBO4* | -11.45 | down |
| ENSG00000214049 | *UCA1* | -5.17 | down |
| ENSG00000142661 | *MYOM3* | -4.22 | down |
| ENSG00000130182 | *ZSCAN10* | -12.31 | down |
| ENSG00000164181 | *ELOVL7* | -4.24 | down |
| ENSG00000293182 | *ENSG00000293182* | -14.06 | down |
| ENSG00000227234 | *SPANXB1* | -7.18 | down |
| ENSG00000249917 | *LINC00536* | -12.18 | down |
| ENSG00000155961 | *RAB39B* | -12.14 | down |
| ENSG00000144821 | *MYH15* | -4.42 | down |
| ENSG00000110203 | *FOLR3* | -6.34 | down |
| ENSG00000286134 | *ENSG00000286134* | -12.11 | down |
| ENSG00000197653 | *DNAH10* | -4.33 | down |
| ENSG00000261644 | *CYLD-AS1* | -11.99 | down |
| ENSG00000175352 | *NRIP3* | -4.95 | down |
| ENSG00000121797 | *CCRL2* | -12.17 | down |
| ENSG00000115602 | *IL1RL1* | -11.20 | down |
| ENSG00000182261 | *NLRP10* | -7.03 | down |
| ENSG00000203721 | *LINC00862* | -4.02 | down |
| ENSG00000060566 | *CREB3L3* | -5.10 | down |
| ENSG00000120279 | *MYCT1* | -12.05 | down |
| ENSG00000168334 | *XIRP1* | -11.01 | down |
| ENSG00000121552 | *CSTA* | -4.94 | down |
| ENSG00000189320 | *FAM180A* | -12.22 | down |
| ENSG00000251381 | *LINC00958* | -5.06 | down |
| ENSG00000100628 | *ASB2* | -6.32 | down |
| ENSG00000188089 | *PLA2G4E* | -10.44 | down |
| ENSG00000230825 | *LINC03016* | -9.93 | down |
| ENSG00000180316 | *PNPLA1* | -4.42 | down |
| ENSG00000283646 | *LINC02009* | -4.13 | down |
| ENSG00000155962 | *CLIC2* | -4.16 | down |
| ENSG00000107249 | *GLIS3* | -4.23 | down |
| ENSG00000232053 | *ENSG00000232053* | -11.59 | down |
| ENSG00000152527 | *PLEKHH2* | -9.95 | down |
| ENSG00000267598 | *ENSG00000267598* | -16.35 | down |
| ENSG00000073756 | *PTGS2* | -10.69 | down |
| ENSG00000148346 | *LCN2* | -4.12 | down |
| ENSG00000258807 | *ENSG00000258807* | -12.21 | down |
| ENSG00000118785 | *SPP1* | -5.69 | down |
| ENSG00000074211 | *PPP2R2C* | -4.34 | down |
| ENSG00000255363 | *LINC02757* | -12.21 | down |
| ENSG00000156265 | *MAP3K7CL* | -6.30 | down |
| ENSG00000197872 | *CYRIA* | -10.52 | down |
| ENSG00000271880 | *ENSG00000271880* | -11.29 | down |
| ENSG00000289384 | *ENSG00000289384* | -12.45 | down |
| ENSG00000124208 | *PEDS1-UBE2V1* | -14.17 | down |
| ENSG00000235385 | *LINC02154* | -12.22 | down |
| ENSG00000164746 | *C7orf57* | -5.21 | down |
| ENSG00000229621 | *LINC01822* | -11.64 | down |
| ENSG00000234155 | *LINC02535* | -5.58 | down |
| ENSG00000164037 | *SLC9B1* | -4.28 | down |
| ENSG00000073737 | *DHRS9* | -6.13 | down |
| ENSG00000236453 | *ENSG00000236453* | -12.60 | down |
| ENSG00000233684 | *LINC01865* | -9.98 | down |
| ENSG00000144583 | *MARCHF4* | -5.08 | down |
| ENSG00000144369 | *FAM171B* | -6.13 | down |
| ENSG00000260729 | *ENSG00000260729* | -4.22 | down |
| ENSG00000104413 | *ESRP1* | -10.29 | down |
| ENSG00000163121 | *NEURL3* | -11.41 | down |
| ENSG00000124731 | *TREM1* | -9.96 | down |
| ENSG00000026751 | *SLAMF7* | -10.34 | down |
| ENSG00000126752 | *SSX1* | -5.79 | down |
| ENSG00000170006 | *TMEM154* | -6.33 | down |
| ENSG00000228626 | *ENSG00000228626* | -7.41 | down |
| ENSG00000230836 | *LINC01293* | -5.60 | down |
| ENSG00000133134 | *BEX2* | -12.37 | down |
| ENSG00000214866 | *DCDC2C* | -11.06 | down |
| ENSG00000163661 | *PTX3* | -6.94 | down |
| ENSG00000189056 | *RELN* | -7.38 | down |
| ENSG00000089127 | *OAS1* | -8.85 | down |
| ENSG00000259867 | *DYNLRB2-AS1* | -9.64 | down |
| ENSG00000204876 | *ENSG00000204876* | -10.20 | down |
| ENSG00000269693 | *ENSG00000269693* | -10.41 | down |
| ENSG00000260836 | *ENSG00000260836* | -11.80 | down |
| ENSG00000256977 | *LIMS3* | -11.01 | down |
| ENSG00000082074 | *FYB1* | -4.02 | down |
| ENSG00000286048 | *ENSG00000286048* | -11.48 | down |
| ENSG00000103154 | *NECAB2* | -10.85 | down |
| ENSG00000235537 | *ENSG00000235537* | -11.57 | down |
| ENSG00000226652 | *PSMD10P2* | -7.03 | down |
| ENSG00000105550 | *FGF21* | -6.76 | down |
| ENSG00000164309 | *CMYA5* | -8.70 | down |
| ENSG00000139318 | *DUSP6* | -6.07 | down |
| ENSG00000125931 | *CITED1* | -4.20 | down |
| ENSG00000149968 | *MMP3* | -8.56 | down |
| ENSG00000289069 | *ENSG00000289069* | -5.74 | down |
| ENSG00000123496 | *IL13RA2* | -11.56 | down |
| ENSG00000228723 | *SRGAP3-AS2* | -11.75 | down |
| ENSG00000291045 | *ENSG00000291045* | -11.56 | down |
| ENSG00000231605 | *LINC01363* | -11.32 | down |
| ENSG00000006128 | *TAC1* | -11.47 | down |
| ENSG00000123610 | *TNFAIP6* | -4.87 | down |
| ENSG00000164266 | *SPINK1* | -11.75 | down |
| ENSG00000251209 | *LINC00923* | -7.34 | down |
| ENSG00000198342 | *ZNF442* | -4.11 | down |
| ENSG00000273415 | *LINC02725* | -5.50 | down |
| ENSG00000019169 | *MARCO* | -11.15 | down |
| ENSG00000197646 | *PDCD1LG2* | -4.67 | down |
| ENSG00000237417 | *XRCC6P1* | -11.39 | down |
| ENSG00000122877 | *EGR2* | -10.33 | down |
| ENSG00000183580 | *FBXL7* | -9.93 | down |
| ENSG00000175344 | *CHRNA7* | -4.57 | down |
| ENSG00000116183 | *PAPPA2* | -8.67 | down |
| ENSG00000198963 | *RORB* | -8.81 | down |
| ENSG00000288758 | *ENSG00000288758* | -5.34 | down |
| ENSG00000104177 | *MYEF2* | -8.22 | down |
| ENSG00000188100 | *FAM25A* | -13.51 | down |
| ENSG00000231298 | *MANCR* | -10.39 | down |
| ENSG00000158270 | *COLEC12* | -9.16 | down |
| ENSG00000157680 | *DGKI* | -4.32 | down |
| ENSG00000161270 | *NPHS1* | -9.19 | down |
| ENSG00000226312 | *CFLAR-AS1* | -9.69 | down |
| ENSG00000170989 | *S1PR1* | -9.60 | down |
| ENSG00000235899 | *LINC01564* | -10.71 | down |
| ENSG00000154553 | *PDLIM3* | -4.89 | down |
| ENSG00000134258 | *VTCN1* | -10.29 | down |
| ENSG00000288951 | *ENSG00000288951* | -10.57 | down |
| ENSG00000129450 | *SIGLEC9* | -10.51 | down |
| ENSG00000151790 | *TDO2* | -9.44 | down |
| ENSG00000122133 | *PAEP* | -11.28 | down |
| ENSG00000139988 | *RDH12* | -10.64 | down |
| ENSG00000162641 | *AKNAD1* | -5.12 | down |
| ENSG00000225106 | *ENSG00000225106* | -11.35 | down |
| ENSG00000174460 | *ZCCHC12* | -10.75 | down |
| ENSG00000188620 | *HMX3* | -10.33 | down |
| ENSG00000158486 | *DNAH3* | -7.87 | down |
| ENSG00000287538 | *ENSG00000287538* | -11.34 | down |
| ENSG00000182901 | *RGS7* | -6.20 | down |
| ENSG00000224239 | *ENSG00000224239* | -12.78 | down |
| ENSG00000154589 | *LY96* | -12.56 | down |
| ENSG00000099937 | *SERPIND1* | -10.53 | down |
| ENSG00000138759 | *FRAS1* | -7.50 | down |
| ENSG00000159495 | *TGM7* | -10.55 | down |
| ENSG00000292995 | *TPRXL* | -9.82 | down |
| ENSG00000148680 | *HTR7* | -6.59 | down |
| ENSG00000173389 | *IQCF1* | -4.43 | down |
| ENSG00000255556 | *ENSG00000255556* | -11.08 | down |
| ENSG00000205097 | *FRG2* | -10.71 | down |
| ENSG00000154263 | *ABCA10* | -8.54 | down |
| ENSG00000111181 | *SLC6A12* | -9.25 | down |
| ENSG00000228538 | *ENSG00000228538* | -11.08 | down |
| ENSG00000067842 | *ATP2B3* | -8.32 | down |
| ENSG00000128253 | *RFPL2* | -10.06 | down |
| ENSG00000189001 | *SBSN* | -10.61 | down |
| ENSG00000282855 | *ENSG00000282855* | -5.27 | down |
| ENSG00000113578 | *FGF1* | -9.32 | down |
| ENSG00000006210 | *CX3CL1* | -9.15 | down |
| ENSG00000286975 | *ENSG00000286975* | -5.46 | down |
| ENSG00000267505 | *ENSG00000267505* | -12.58 | down |
| ENSG00000164400 | *CSF2* | -12.10 | down |
| ENSG00000285722 | *ENSG00000285722* | -10.36 | down |
| ENSG00000080031 | *PTPRH* | -4.19 | down |
| ENSG00000214407 | *RDUR* | -10.53 | down |
| ENSG00000145451 | *GLRA3* | -4.41 | down |
| ENSG00000204091 | *TDRG1* | -10.00 | down |
| ENSG00000064886 | *CHI3L2* | -9.85 | down |
| ENSG00000180777 | *ANKRD30B* | -8.82 | down |
| ENSG00000132185 | *FCRLA* | -9.83 | down |
| ENSG00000250303 | *LINC02762* | -8.47 | down |
| ENSG00000288783 | *ENSG00000288783* | -12.31 | down |
| ENSG00000258791 | *LINC00520* | -4.64 | down |
| ENSG00000137090 | *DMRT1* | -9.92 | down |
| ENSG00000163827 | *LRRC2* | -9.10 | down |
| ENSG00000225746 | *MEG8* | -6.52 | down |
| ENSG00000245008 | *ENSG00000245008* | -9.25 | down |
| ENSG00000215267 | *AKR1C7P* | -11.67 | down |
| ENSG00000285898 | *ENSG00000285898* | -10.20 | down |
| ENSG00000018625 | *ATP1A2* | -4.57 | down |
| ENSG00000228526 | *MIR34AHG* | -6.00 | down |
| ENSG00000260186 | *LINC02137* | -10.50 | down |
| ENSG00000106178 | *CCL24* | -10.84 | down |
| ENSG00000101670 | *LIPG* | -4.83 | down |
| ENSG00000272975 | *MYHAS* | -4.05 | down |
| ENSG00000125675 | *GRIA3* | -8.67 | down |
| ENSG00000240476 | *LINC00973* | -4.49 | down |
| ENSG00000155926 | *SLA* | -4.32 | down |
| ENSG00000241749 | *RPSAP52* | -11.71 | down |
| ENSG00000249026 | *CTNNA1P1* | -5.74 | down |
| ENSG00000172818 | *OVOL1* | -9.49 | down |
| ENSG00000143333 | *RGS16* | -10.10 | down |
| ENSG00000164122 | *ASB5* | -4.25 | down |
| ENSG00000227262 | *HCG4B* | -11.29 | down |
| ENSG00000258433 | *ENSG00000258433* | -10.72 | down |
| ENSG00000166664 | *CHRFAM7A* | -4.27 | down |
| ENSG00000107611 | *CUBN* | -7.56 | down |
| ENSG00000184459 | *BPIFC* | -10.06 | down |
| ENSG00000125538 | *IL1B* | -4.45 | down |
| ENSG00000243742 | *RPLP0P2* | -5.27 | down |
| ENSG00000260765 | *CES1P2* | -4.75 | down |
| ENSG00000203837 | *PNLIPRP3* | -10.03 | down |
| ENSG00000250978 | *ENSG00000250978* | -11.47 | down |
| ENSG00000186472 | *PCLO* | -5.85 | down |
| ENSG00000101280 | *ANGPT4* | -8.95 | down |
| ENSG00000104808 | *DHDH* | -11.02 | down |
| ENSG00000187753 | *C9orf153* | -9.71 | down |
| ENSG00000101850 | *GPR143* | -9.95 | down |
| ENSG00000166317 | *SYNPO2L* | -5.23 | down |
| ENSG00000289313 | *LINC01512* | -9.99 | down |
| ENSG00000082126 | *MPP4* | -4.43 | down |
| ENSG00000225899 | *FRG2B* | -10.13 | down |
| ENSG00000177103 | *DSCAML1* | -8.26 | down |
| ENSG00000163207 | *IVL* | -10.02 | down |
| ENSG00000268223 | *ARL14EPL* | -10.10 | down |
| ENSG00000273669 | *ENSG00000273669* | -11.66 | down |
| ENSG00000153303 | *FRMD1* | -7.87 | down |
| ENSG00000234492 | *RPL34-DT* | -8.54 | down |
| ENSG00000164082 | *GRM2* | -4.36 | down |
| ENSG00000243627 | *SMIM34* | -11.01 | down |
| ENSG00000284624 | *ENSG00000284624* | -4.42 | down |
| ENSG00000257642 | *C12orf75-AS1* | -11.86 | down |
| ENSG00000133454 | *MYO18B* | -7.78 | down |
| ENSG00000290241 | *ENSG00000290241* | -10.41 | down |
| ENSG00000120903 | *CHRNA2* | -8.78 | down |
| ENSG00000235643 | *LINC01647* | -4.55 | down |
| ENSG00000145362 | *ANK2* | -4.14 | down |
| ENSG00000136928 | *GABBR2* | -8.05 | down |
| ENSG00000287796 | *ENSG00000287796* | -9.08 | down |
| ENSG00000140968 | *IRF8* | -7.94 | down |
| ENSG00000251410 | *ENSG00000251410* | -9.00 | down |
| ENSG00000244040 | *IL12A-AS1* | -7.34 | down |
| ENSG00000181355 | *OFCC1* | -9.38 | down |
| ENSG00000198400 | *NTRK1* | -8.43 | down |
| ENSG00000109511 | *ANXA10* | -4.04 | down |
| ENSG00000180353 | *HCLS1* | -8.50 | down |
| ENSG00000114771 | *AADAC* | -10.23 | down |
| ENSG00000103546 | *SLC6A2* | -7.93 | down |
| ENSG00000251273 | *LINC02228* | -8.65 | down |
| ENSG00000223812 | *PYDC2-AS1* | -4.89 | down |
| ENSG00000173077 | *DELEC1* | -8.09 | down |
| ENSG00000113905 | *HRG* | -8.57 | down |
| ENSG00000250697 | *ENSG00000250697* | -9.08 | down |
| ENSG00000234509 | *SOD1-DT* | -9.65 | down |
| ENSG00000267405 | *ENSG00000267405* | -4.09 | down |
| ENSG00000258096 | *SLC38A2-AS1* | -4.49 | down |
| ENSG00000223638 | *RFPL4A* | -10.57 | down |
| ENSG00000109927 | *TECTA* | -7.72 | down |
| ENSG00000012779 | *ALOX5* | -8.98 | down |
| ENSG00000287516 | *ENSG00000287516* | -7.43 | down |
| ENSG00000287064 | *ENSG00000287064* | -4.03 | down |
| ENSG00000287277 | *ENSG00000287277* | -6.41 | down |
| ENSG00000261210 | *CLEC19A* | -8.99 | down |
| ENSG00000232949 | *ENSG00000232949* | -9.64 | down |
| ENSG00000143954 | *REG3G* | -9.90 | down |
| ENSG00000188916 | *INSYN2A* | -8.53 | down |
| ENSG00000293142 | *ENSG00000293142* | -4.02 | down |
| ENSG00000227790 | *SPECC1P1* | -5.06 | down |
| ENSG00000278961 | *ENSG00000278961* | -10.64 | down |
| ENSG00000293176 | *RPSAP52* | -6.08 | down |
| ENSG00000157782 | *CABP1* | -4.07 | down |
| ENSG00000105088 | *OLFM2* | -9.43 | down |
| ENSG00000125355 | *TMEM255A* | -8.72 | down |
| ENSG00000286042 | *LCAL1* | -8.52 | down |
| ENSG00000103316 | *CRYM* | -4.10 | down |
| ENSG00000289497 | *ENSG00000289497* | -10.28 | down |
| ENSG00000235119 | *ENSG00000235119* | -13.18 | down |
| ENSG00000165694 | *FRMD7* | -8.87 | down |
| ENSG00000259518 | *LINC01583* | -4.26 | down |
| ENSG00000293503 | *ENSG00000293503* | -10.93 | down |
| ENSG00000111704 | *NANOG* | -8.17 | down |
| ENSG00000251537 | *ENSG00000251537* | -6.87 | down |
| ENSG00000255446 | *ENSG00000255446* | -11.57 | down |
| ENSG00000261780 | *LINC02582* | -9.39 | down |
| ENSG00000198216 | *CACNA1E* | -6.20 | down |
| ENSG00000183778 | *B3GALT5* | -6.74 | down |
| ENSG00000238120 | *LINC01589* | -4.16 | down |
| ENSG00000276975 | *HYDIN2* | -6.81 | down |
| ENSG00000253161 | *LINC01605* | -9.46 | down |
| ENSG00000006788 | *MYH13* | -4.23 | down |
| ENSG00000224259 | *LINC01133* | -8.62 | down |
| ENSG00000151136 | *ABTB3* | -7.61 | down |
| ENSG00000261104 | *ENSG00000261104* | -4.17 | down |
| ENSG00000231738 | *TSPAN19* | -8.56 | down |
| ENSG00000291288 | *ENSG00000291288* | -4.19 | down |
| ENSG00000129244 | *ATP1B2* | -8.76 | down |
| ENSG00000255395 | *ENSG00000255395* | -11.44 | down |
| ENSG00000166589 | *CDH16* | -8.80 | down |
| ENSG00000267193 | *ENSG00000267193* | -9.55 | down |
| ENSG00000175820 | *CCDC168* | -6.14 | down |
| ENSG00000152661 | *GJA1* | -8.80 | down |
| ENSG00000215182 | *MUC5AC* | -6.53 | down |
| ENSG00000066032 | *CTNNA2* | -7.66 | down |
| ENSG00000251127 | *ENSG00000251127* | -9.12 | down |
| ENSG00000174358 | *SLC6A19* | -8.15 | down |
| ENSG00000236507 | *ENSG00000236507* | -8.60 | down |
| ENSG00000260498 | *ENSG00000260498* | -10.06 | down |
| ENSG00000205420 | *KRT6A* | -9.02 | down |
| ENSG00000167083 | *GNGT2* | -9.76 | down |
| ENSG00000237153 | *ENSG00000237153* | -4.31 | down |
| ENSG00000172867 | *KRT2* | -9.11 | down |
| ENSG00000272094 | *ENSG00000272094* | -10.65 | down |
| ENSG00000162891 | *IL20* | -10.10 | down |
| ENSG00000214447 | *FAM187A* | -7.04 | down |
| ENSG00000170925 | *TEX13B* | -10.29 | down |
| ENSG00000144481 | *TRPM8* | -7.41 | down |
| ENSG00000100055 | *CYTH4* | -4.01 | down |
| ENSG00000250497 | *ENSG00000250497* | -8.27 | down |
| ENSG00000169551 | *CT55* | -9.77 | down |
| ENSG00000289873 | *ENSG00000289873* | -11.01 | down |
| ENSG00000293037 | *ENSG00000293037* | -9.86 | down |
| ENSG00000211899 | *IGHM* | -9.55 | down |
| ENSG00000158125 | *XDH* | -5.88 | down |
| ENSG00000064787 | *BCAS1* | -7.05 | down |
| ENSG00000183813 | *CCR4* | -8.84 | down |
| ENSG00000238193 | *HADHBP1* | -9.95 | down |
| ENSG00000169213 | *RAB3B* | -5.10 | down |
| ENSG00000163803 | *PLB1* | -4.20 | down |
| ENSG00000101605 | *MYOM1* | -7.66 | down |
| ENSG00000254423 | *ENSG00000254423* | -10.20 | down |
| ENSG00000085563 | *ABCB1* | -7.73 | down |
| ENSG00000175658 | *DRD5P2* | -9.36 | down |
| ENSG00000125735 | *TNFSF14* | -8.11 | down |
| ENSG00000274060 | *MIR6724-2* | -6.47 | down |
| ENSG00000162398 | *CIMAP2* | -9.58 | down |
| ENSG00000290060 | *ENSG00000290060* | -10.35 | down |
| ENSG00000165731 | *RET* | -7.25 | down |
| ENSG00000122547 | *EEPD1* | -5.29 | down |
| ENSG00000174844 | *DNAH12* | -6.66 | down |
| ENSG00000256083 | *ENSG00000256083* | -11.48 | down |
| ENSG00000198729 | *PPP1R14C* | -9.24 | down |
| ENSG00000289850 | *ENSG00000289850* | -11.29 | down |
| ENSG00000184647 | *PRSS55* | -8.74 | down |
| ENSG00000231811 | *ENSG00000231811* | -8.51 | down |
| ENSG00000137491 | *SLCO2B1* | -6.80 | down |
| ENSG00000160862 | *AZGP1* | -4.35 | down |
| ENSG00000197506 | *SLC28A3* | -8.01 | down |
| ENSG00000289086 | *ENSG00000289086* | -10.99 | down |
| ENSG00000171533 | *MAP6* | -7.60 | down |
| ENSG00000214121 | *PRDX1P1* | -11.13 | down |
| ENSG00000215009 | *ACSM4* | -8.90 | down |
| ENSG00000265728 | *ENSG00000265728* | -10.51 | down |
| ENSG00000139151 | *PLCZ1* | -8.51 | down |
| ENSG00000236393 | *LINC03099* | -8.56 | down |
| ENSG00000112936 | *C7* | -6.94 | down |
| ENSG00000288952 | *ENSG00000288952* | -10.04 | down |
| ENSG00000282826 | *FRG1CP* | -11.05 | down |
| ENSG00000283265 | *ENSG00000283265* | -4.04 | down |
| ENSG00000204632 | *HLA-G* | -5.25 | down |
| ENSG00000140557 | *ST8SIA2* | -7.66 | down |
| ENSG00000196188 | *CTSE* | -8.42 | down |
| ENSG00000258599 | *ENSG00000258599* | -11.04 | down |
| ENSG00000287454 | *ENSG00000287454* | -7.97 | down |
| ENSG00000287763 | *ENSG00000287763* | -10.16 | down |
| ENSG00000081051 | *AFP* | -8.61 | down |
| ENSG00000272666 | *KLHDC7B-DT* | -4.82 | down |
| ENSG00000288710 | *ENSG00000288710* | -5.63 | down |
| ENSG00000286535 | *ENSG00000286535* | -8.87 | down |
| ENSG00000203786 | *KPRP* | -8.87 | down |
| ENSG00000104415 | *CCN4* | -7.90 | down |
| ENSG00000230951 | *GPS2P2* | -10.49 | down |
| ENSG00000287687 | *ENSG00000287687* | -9.29 | down |
| ENSG00000214815 | *IMPDH1P3* | -9.58 | down |
| ENSG00000257743 | *MGAM2* | -7.60 | down |
| ENSG00000185972 | *CCIN* | -9.31 | down |
| ENSG00000187094 | *CCK* | -4.43 | down |
| ENSG00000173366 | *ENSG00000173366* | -8.17 | down |
| ENSG00000114805 | *PLCH1* | -4.62 | down |
| ENSG00000172139 | *SLC9C1* | -8.09 | down |
| ENSG00000104327 | *CALB1* | -7.24 | down |
| ENSG00000272508 | *ENSG00000272508* | -9.86 | down |
| ENSG00000287740 | *ENSG00000287740* | -9.26 | down |
| ENSG00000258770 | *LINC02330* | -10.95 | down |
| ENSG00000206585 | *RNVU1-7* | -12.74 | down |
| ENSG00000122641 | *INHBA* | -7.00 | down |
| ENSG00000196632 | *WNK3* | -6.66 | down |
| ENSG00000165702 | *GFI1B* | -4.13 | down |
| ENSG00000135917 | *SLC19A3* | -4.11 | down |
| ENSG00000248431 | *ENSG00000248431* | -8.68 | down |
| ENSG00000185903 | *OR11N1P* | -10.28 | down |
| ENSG00000165125 | *TRPV6* | -6.46 | down |
| ENSG00000171094 | *ALK* | -7.64 | down |
| ENSG00000288002 | *ENSG00000288002* | -8.53 | down |
| ENSG00000275950 | *MIR6724-1* | -6.01 | down |
| ENSG00000236056 | *GAPDHP14* | -10.21 | down |
| ENSG00000146469 | *VIP* | -9.51 | down |
| ENSG00000165805 | *C12orf50* | -7.96 | down |
| ENSG00000127152 | *BCL11B* | -6.97 | down |
| ENSG00000233347 | *ERP29P1* | -10.55 | down |
| ENSG00000289029 | *ENSG00000289029* | -9.71 | down |
| ENSG00000099954 | *CECR2* | -4.44 | down |
| ENSG00000143502 | *SUSD4* | -7.64 | down |
| ENSG00000198574 | *SH2D1B* | -8.58 | down |
| ENSG00000136689 | *IL1RN* | -8.11 | down |
| ENSG00000268812 | *LIF-AS2* | -10.72 | down |
| ENSG00000254545 | *ENSG00000254545* | -4.08 | down |
| ENSG00000177752 | *YIPF7* | -7.85 | down |
| ENSG00000158022 | *TRIM63* | -8.62 | down |
| ENSG00000262429 | *ENSG00000262429* | -11.05 | down |
| ENSG00000168955 | *TM4SF20* | -8.42 | down |
| ENSG00000230221 | *ENSG00000230221* | -8.70 | down |
| ENSG00000133083 | *DCLK1* | -4.61 | down |
| ENSG00000100365 | *NCF4* | -9.07 | down |
| ENSG00000168143 | *FAM83B* | -7.72 | down |
| ENSG00000212724 | *KRTAP2-3* | -10.64 | down |
| ENSG00000243781 | *ENSG00000243781* | -10.41 | down |
| ENSG00000183347 | *GBP6* | -7.78 | down |
| ENSG00000118432 | *CNR1* | -7.23 | down |
| ENSG00000158458 | *NRG2* | -7.74 | down |
| ENSG00000235748 | *SEPTIN14P12* | -10.78 | down |
| ENSG00000080493 | *SLC4A4* | -7.18 | down |
| ENSG00000113763 | *UNC5A* | -8.25 | down |
| ENSG00000109944 | *JHY* | -6.89 | down |
| ENSG00000251179 | *TMEM92-AS1* | -10.32 | down |
| ENSG00000116745 | *RPE65* | -7.83 | down |
| ENSG00000142611 | *PRDM16* | -6.60 | down |
| ENSG00000174611 | *KY* | -6.75 | down |
| ENSG00000223443 | *USP17L2* | -8.66 | down |
| ENSG00000281991 | *TMEM265* | -5.59 | down |
| ENSG00000204363 | *SPANXN5* | -10.54 | down |
| ENSG00000228251 | *ENSG00000228251* | -10.89 | down |
| ENSG00000144908 | *ALDH1L1* | -6.38 | down |
| ENSG00000251600 | *GUSBP5* | -6.03 | down |
| ENSG00000254560 | *BBOX1-AS1* | -9.88 | down |
| ENSG00000185306 | *C12orf56* | -7.47 | down |
| ENSG00000177575 | *CD163* | -7.70 | down |
| ENSG00000205918 | *PDPK2P* | -10.48 | down |
| ENSG00000285774 | *SAMD4A-AS1* | -8.32 | down |
| ENSG00000259590 | *LINC02244* | -11.55 | down |
| ENSG00000257060 | *ENSG00000257060* | -7.05 | down |
| ENSG00000258077 | *ENSG00000258077* | -9.46 | down |
| ENSG00000288556 | *ENSG00000288556* | -9.77 | down |
| ENSG00000160219 | *GAB3* | -7.84 | down |
| ENSG00000131738 | *KRT33B* | -9.55 | down |
| ENSG00000237596 | *PDE7B-AS1* | -4.90 | down |
| ENSG00000183801 | *OLFML1* | 4.12 | up |
| ENSG00000188517 | *COL25A1* | 4.62 | up |
| ENSG00000215472 | *RPL17-C18orf32* | 15.40 | up |
| ENSG00000285547 | *ENSG00000285547* | 12.71 | up |
| ENSG00000226792 | *C13orf42* | 4.25 | up |
| ENSG00000257921 | *ENSG00000257921* | 13.77 | up |
| ENSG00000261796 | *ISY1-RAB43* | 12.70 | up |
| ENSG00000153292 | *ADGRF1* | 9.13 | up |
| ENSG00000173626 | *TRAPPC3L* | 11.35 | up |
| ENSG00000285238 | *ENSG00000285238* | 12.09 | up |
| ENSG00000274049 | *INO80B-WBP1* | 13.16 | up |
| ENSG00000135697 | *BCO1* | 6.00 | up |
| ENSG00000288622 | *PDCD6-AHRR* | 7.60 | up |
| ENSG00000278982 | *ENSG00000278982* | 11.43 | up |
| ENSG00000256349 | *ENSG00000256349* | 10.75 | up |
| ENSG00000214248 | *ENSG00000214248* | 10.27 | up |
| ENSG00000269955 | *FMC1-LUC7L2* | 11.36 | up |
| ENSG00000258586 | *LINC02274* | 5.26 | up |
| ENSG00000266573 | *ENSG00000266573* | 8.71 | up |
| ENSG00000236197 | *PPP1R9A-AS1* | 9.75 | up |
| ENSG00000283405 | *ENSG00000283405* | 4.27 | up |
| ENSG00000227630 | *LINC01132* | 9.35 | up |
| ENSG00000109101 | *FOXN1* | 9.73 | up |
| ENSG00000264717 | *NPY4R2* | 4.22 | up |
| ENSG00000235142 | *LINC02532* | 7.92 | up |
| ENSG00000261335 | *ENSG00000261335* | 4.94 | up |
| ENSG00000133980 | *VRTN* | 9.55 | up |
| ENSG00000149575 | *SCN2B* | 9.05 | up |
| ENSG00000132872 | *SYT4* | 5.09 | up |
| ENSG00000109906 | *ZBTB16* | 7.21 | up |
| ENSG00000078319 | *PMS2P1* | 5.16 | up |
| ENSG00000234949 | *RAB17-DT* | 10.46 | up |
| ENSG00000114113 | *RBP2* | 4.72 | up |
| ENSG00000164530 | *PI16* | 9.98 | up |
| ENSG00000290891 | *ENSG00000290891* | 10.09 | up |
| ENSG00000260139 | *CSPG4P13* | 4.06 | up |
| ENSG00000289840 | *ENSG00000289840* | 4.39 | up |
| ENSG00000122194 | *PLG* | 7.81 | up |
| ENSG00000267780 | *ENSG00000267780* | 11.90 | up |
| ENSG00000095587 | *TLL2* | 5.01 | up |
| ENSG00000119782 | *FKBP1B* | 10.13 | up |
| ENSG00000144648 | *ACKR2* | 8.38 | up |
| ENSG00000244122 | *UGT1A7* | 4.51 | up |
| ENSG00000104044 | *OCA2* | 4.33 | up |
| ENSG00000286570 | *ENSG00000286570* | 4.52 | up |
| ENSG00000259772 | *LINC03034* | 4.17 | up |
| ENSG00000250362 | *ENSG00000250362* | 10.99 | up |
| ENSG00000111913 | *RIPOR2* | 7.47 | up |
| ENSG00000179772 | *FOXS1* | 4.13 | up |
| ENSG00000188162 | *OTOG* | 7.27 | up |
| ENSG00000231131 | *LNCAROD* | 8.99 | up |
| ENSG00000118194 | *TNNT2* | 7.84 | up |
| ENSG00000070526 | *ST6GALNAC1* | 8.93 | up |
| ENSG00000180539 | *LINC02908* | 8.22 | up |
| ENSG00000255092 | *ENSG00000255092* | 11.35 | up |
| ENSG00000264177 | *ENSG00000264177* | 8.20 | up |
| ENSG00000229388 | *TAF12-DT* | 4.05 | up |
| ENSG00000117983 | *MUC5B* | 4.29 | up |
| ENSG00000245651 | *ENSG00000245651* | 4.09 | up |
| ENSG00000265254 | *ENSG00000265254* | 11.46 | up |
| ENSG00000113361 | *CDH6* | 6.80 | up |
| ENSG00000257771 | *LINC02395* | 7.18 | up |
| ENSG00000262179 | *MYMX* | 10.61 | up |
| ENSG00000116039 | *ATP6V1B1* | 9.26 | up |
| ENSG00000178803 | *ADORA2A-AS1* | 8.26 | up |
| ENSG00000290024 | *ENSG00000290024* | 11.50 | up |
| ENSG00000168748 | *CA7* | 9.83 | up |
| ENSG00000286070 | *ENSG00000286070* | 10.93 | up |
| ENSG00000246548 | *LINC02288* | 8.72 | up |
| ENSG00000231982 | *MCF2L2P1* | 11.85 | up |
| ENSG00000280182 | *ENSG00000280182* | 8.98 | up |
| ENSG00000289894 | *ENSG00000289894* | 9.48 | up |
| ENSG00000291025 | *ENSG00000291025* | 7.52 | up |
| ENSG00000236212 | *ENSG00000236212* | 9.49 | up |
| ENSG00000260296 | *ENSG00000260296* | 9.97 | up |
| ENSG00000273088 | *ENSG00000273088* | 10.98 | up |
| ENSG00000166342 | *NETO1* | 7.05 | up |
| ENSG00000269994 | *LINC02893* | 8.77 | up |
| ENSG00000255471 | *PRSS23-AS1* | 4.09 | up |
| ENSG00000215493 | *ENSG00000215493* | 4.11 | up |
| ENSG00000260388 | *LINC00562* | 4.59 | up |
| ENSG00000289019 | *ENSG00000289019* | 10.33 | up |
| ENSG00000234177 | *LINC01114* | 8.14 | up |
| ENSG00000242798 | *ENSG00000242798* | 10.81 | up |
| ENSG00000289746 | *TARP* | 10.60 | up |
| ENSG00000291338 | *ENSG00000291338* | 9.25 | up |
| ENSG00000178175 | *ZNF366* | 7.37 | up |
| ENSG00000232470 | *ENSG00000232470* | 11.30 | up |
| ENSG00000287952 | *ENSG00000287952* | 9.23 | up |
| ENSG00000254548 | *ENSG00000254548* | 11.79 | up |
| ENSG00000261934 | *PCDHGA9* | 8.07 | up |
| ENSG00000139445 | *FOXN4* | 8.13 | up |
| ENSG00000270757 | *HSPE1-MOB4* | 15.04 | up |
| ENSG00000260468 | *LINC01290* | 10.80 | up |

## Supplementary Figures


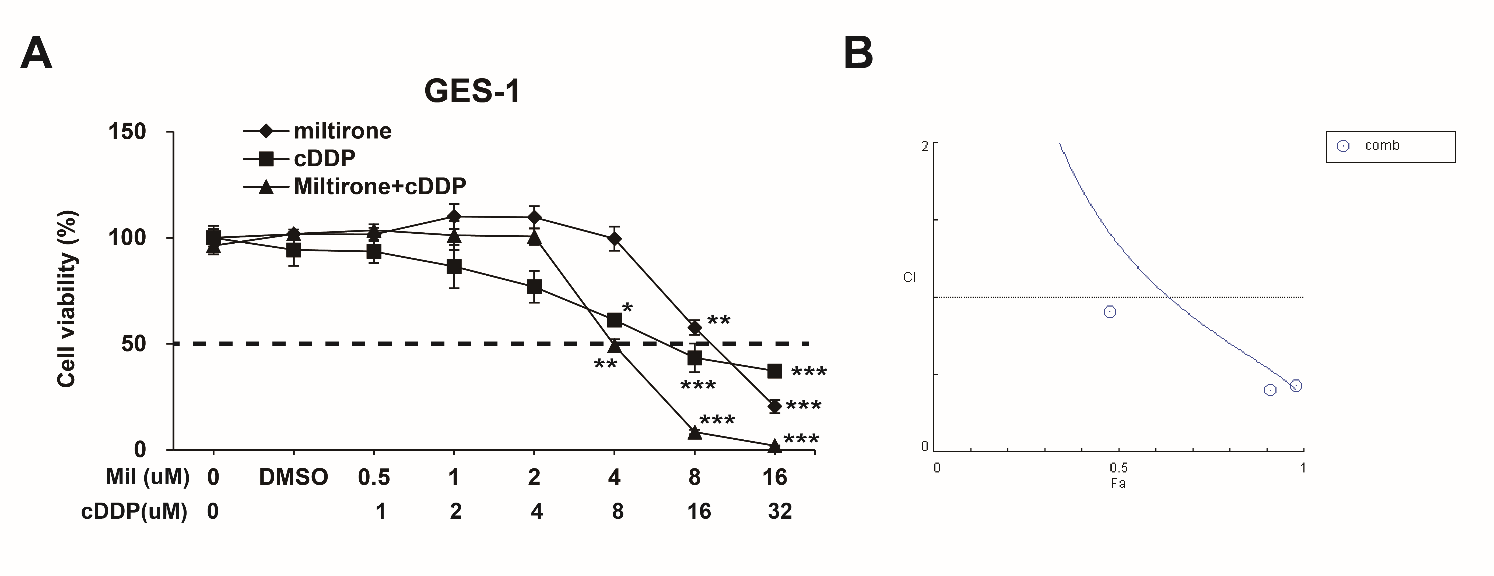


Supplementary Figure S1, The inhibitory effect of miltirone, cisplatin, and combination therapy on the proliferation of one normal gastric mucosal epithelial cell line (GES-1).

(A) Miltirone, cisplatin, and combination treatment inhibited the proliferation of GES-1 as shown by decreased cell viability. The drug concentration-cell viability curve was generated as a percentage of viable cells. (B) The synergistic effect between miltirone and cisplatin is represented by a Fa-CI diagram. The above data are from three replicate experiments (mean ± SD). Compared with the control group, *p < 0.05, **p < 0.01 or ***p < 0.001.


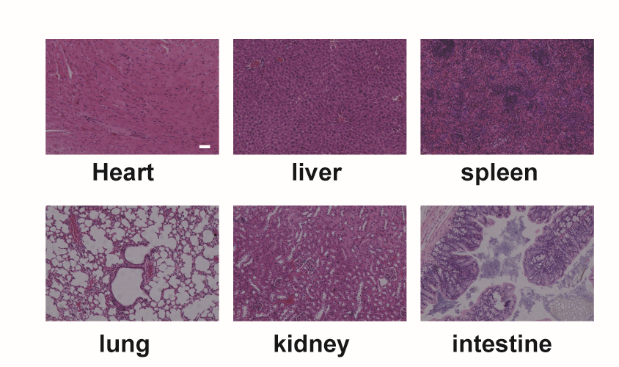


Supplementary Figure S2, H&E staining of the heart, kidney, liver, spleen, lung and intestine from miltirone-treated nude mice.
